# Phenolic and Lignan Glycosides from the Butanol Extract of *Averrhoa carambola* L. Root

**DOI:** 10.3390/molecules171012330

**Published:** 2012-10-19

**Authors:** Qingwei Wen, Xing Lin, Liu Yeqi, Xiaohui Xu, Tao Liang, Ni Zheng, Renbin Huang

**Affiliations:** 1 Pharmaceutical College, Guangxi Medical University; No. 22, Shuangyong Road, Nanning, Guangxi 530021, China; Email: wqw760623@163.com (Q.W.); gxLx60@163.com (X.L.); maskinjogja@yahoo.com.sg (K.); 2 Department of Pediatrics, Louisiana State University Health Sciences Center, the Research Institute for Children Children’s Hospital, 200 Henry Clay Avenue, New Orleans, LA 70118, USA; Email: yeqi_liu@hotmail.com

**Keywords:** phenolic and lignan glycosides, *Averrhoa carambola* L., butanol extract

## Abstract

Fifteen compounds, which included six chiral lignans and nine phenolic glycosides, were separated from the butanol fraction of *Averrhoa carambola L.* root and identified. All of the compounds, namely 3,4,5-trimethoxyphenol-1-*O*-*β*-*D*-glucopyranoside (**1**), benzyl-1-*O- β*-*D*-glucopyranoside (**2**), (+)-5'-methoxyisolariciresinol 3α-*O- β*-*D*-gluco-pyranoside (**3**), (+)-isolariciresinol 3α-*O- β*-*D*-glucopyranoside (**4**), koaburaside (**5**), (+)-lyoniresinol 3α-*O- β*-*D*-glucopyranoside (**6**), (−)-lyoniresinol 3α-*O- β*-*D*-glucopyranoside (**7**), (−)-5'-methoxyisolariciresinol 3α-*O- β*-*D*-glucopyranoside (**8**), (−)-isolariciresinol 3α-*O- β*-*D*-glucopyranoside (**9**), 3,5-dimethoxy-4-hydroxyphenyl 1-*O-β*-apiofuranosyl (1''→6')-*O*-*β*-*D*-glucopyranoside (**10**), 3,4,5-trimethoxyphenyl 1-*O-β*-apiofuranosyl (1''→6')-β-gluco-pyranoside (**11**), methoxyhydroquinone-4*- β -D*-glucopyranoside (**12**), (2*S*)-2-*O- β*-*D*-gluco-pyranosyl-2-hydroxyphenylacetic acid (**13**), 3-hydroxy-4-methoxyphenol 1-*O -β*-*D*-apio-furanosyl-(1''→6')-*O - β*-*D*-glucopyranoside (**14**) and 4-hydroxy-3-methoxyphenol 1-*O -β*-*D*-apiofuranosyl-(1''→6')-*O - β*-*D*-glucopyranoside (**15**) were isolated from this plant for the first time.

## 1. Introduction

Nowdays, herbal medicine is accepted worldwide as an alternative therapy [1,2]. *Averrhoa carambola* L. (Oxalidaceae) is a perennial herb widely distributed in China, Taiwan, Malaysia, India, Brazil, America, *etc.* Its roots, have been used as a Traditional Chinese Medicine for thousands of years in the remedy of lithangiuria, arthralgia and chronic paroxysmal headache. In our previous study, both ethanol extract and polysaccharide from the roots showed hypoglycemic and antioxidant effects [3,4]. Many publications have also indicated that compounds from *Averrhoa carambola* leaves displayed hypoglycemic, hypotriglyceridemic, anti-lipid peroxidative and anti-atherogenic properties in streptozocin-induced diabetic rats [5,6].

Previous literature reports on *Averrhoa carambola* L. have only reported the isolation and identification of a few compounds such as β-sitosterol, lupeol and 1,5-dihydroxy-6,7-dimethoxy-2-methyl-anthraquinone 3-*O*-β-glucopyranoside [7,8,9,10]. Therefore, this study involved separating more compounds from the herb aiming to offer better insight into its chemical constituents. In this research, the 60% aqueous ethanol (aq. EtOH) extract from *Averrhoa carambola* L*.* roots was suspended in H_2_O and further extracted with cyclohexane, ethyl acetate (EtOAc) and *n*-butanol (*n*-BuOH), respectively. Then, the butanol extract was successively purified by open silica gel, Sephadex LH-20, ODS column and P-HPLC to obtain 15 compounds that could be useful for investigating the hypoglycemic or antioxidant substances of the plant and standardization of the butanol extract from this herb in the next step.

## 2. Results and Discussion

**Figure 1:**
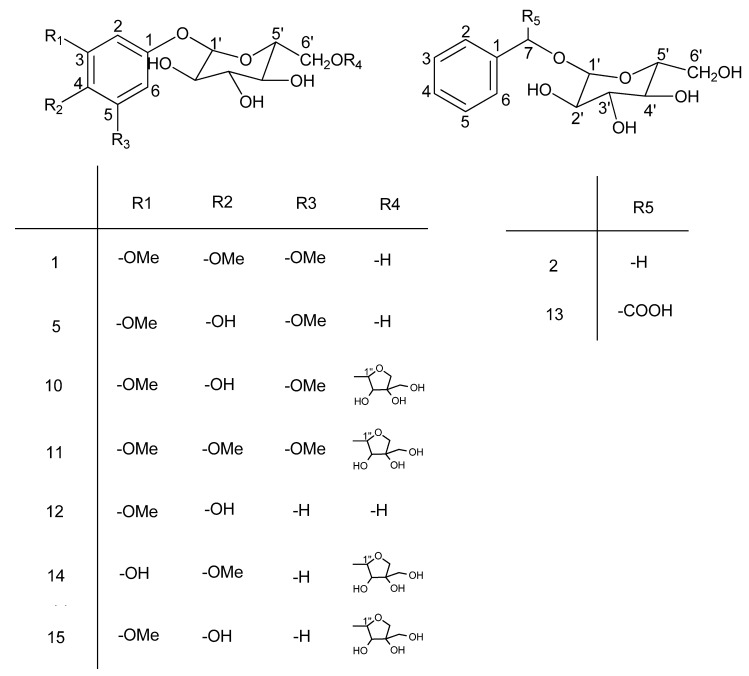
Structures of compounds **1**, **2**, **5** and **10**–**15**.

**Figure 2:**
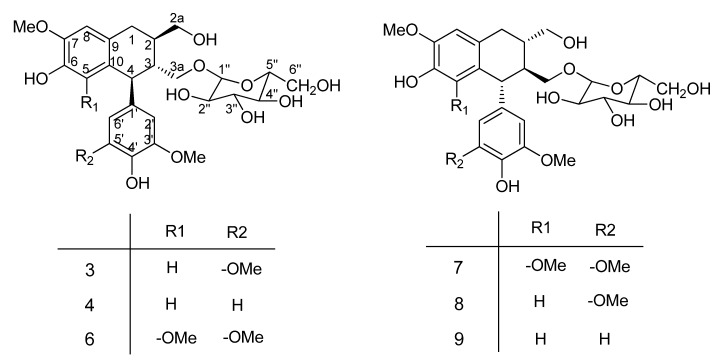
Structures of compounds **3**, **4** and **6**–**9**.

The 60% aq. EtOH extract of *Averrhoa carambola* L. root was successively separated by cyclohexane, EtOAc and *n*-BuOH. From the n-BuOH fraction, fifteen known compounds (1–15, listed in the Abstract, structures shown on Figure 1 and Figure 2) were isolated and their structures confirmed by detailed FTIR, NMR (^1^H, ^13^C) data comparison with those in the literature [11,12,13,14,15,16,17,18,19,20,21,22]. ^13^C-NMR data of the compounds is summarized in Table 1 and Table 2. ^1^H-NMR data is given in the Experimental. These nine phenolic and six lignan glycosides still not been previously isolated and identified from this plant. Just as mentioned in the Introduction, Our preliminary investigations have shown that the ethanolic extract of *Averrhoa carambola L.* root could relieve the lipid peroxide reactions and has a significant hypoglycemic effect in the streptozotocin-induced diabetic mice, suggesting it may be a potential hypoglycemic agent for the treatment of diabetes and its complications [3]. Compounds 6 (418.9 mg) and 7 (534.7 mg), as the main chemical ingredients of the *n*-BuOH extract, may play a key role in the observed anti-diabetic effect. To explore the active substances in this plant, these two compounds’ hypoglycemic activities will be investigated in the near future. The structures of the compounds are shown on Figure 1 and Figure 2.

**Table 1:** ^13^C-NMR data of compounds **1**, **2**, **5** and **10**–**15** (150 MHz, MeOD).

C	1	2	5	10	11	12	13	14	15
**1**	156.3	139.1	156.0	156.0	134.7	152.9	138.8	152.7	155.0
**2**	96.2	129.3	94.6	94.6	96.3	103.7	129.3	105.2	104.4
**3**	154.8	129.2	154.8	154.9	154.9	149.3	129.3	149.3	152.1
**4**	145.8	128.7	129.7	129.5	155.9	142.8	128.5	143.0	141.1
**5**	154.8	129.2	154.8	154.9	154.9	116.0	129.3	116.3	120.7
**6**	96.2	129.3	94.6	94.6	96.3	110.0	129.3	110.1	111.0
**7**	-	71.7	-	-	-	-	81.4	-	-
**R1**	56.6	-	56.8	56.8	56.8	56.4	-	-	56.6
**R2**	61.2	-	-	-	61.3	-	-	56.5	-
**R3**	56.6	-	56.8	56.8	56.8	-	-	-	-
**R5**	-	-	-		-	-	176.5	-	-
**1'**	103.2	103.3	106.2	105.1	103.0	103.7	103.5	103.6	104.4
**2'**	75.0	75.2	75.8	74.9	74.9	75.0	75.1	74.9	75.0
**3'**	78.2	78.0	78.3	77.8	77.2	78.2	78.3	77.7	78.1
**4'**	71.7	71.8	71.4	71.3	71.5	71.6	71.4	71.4	71.7
**5'**	78.4	78.0	77.8	75.6	77.8	78.1	78.0	77.3	77.0
**6'**	61.2	62.8	62.6	69.3	67.0	62.5	62.6	70.1	68.7
**1''**	-	-	-	105.9	105.9	-	-	103.9	111.0
**2''**	-	-	-	77.5	77.2	-	-	77.3	77.8
**3''**	-	-	-	77.8	77.8	-	-	77.9	80.6
**4''**	-	-	-	74.9	74.9	-	-	74.9	75.1
**5''**	-	-	-	66.8	61.3	-	-	66.8	65.7

**Table 2:** ^13^C-NMR data of compounds **3**, **4** and **6**–**9** (150 MHz, MeOD).

C	3 *	4	6	7	8	9
**1**	33.7	33.8	33.8	33.8	33.6	33.6
**2**	39.1	39.6	40.6	41.3	41.1	41.1
**2a**	64.6	64.9	66.2	66.2	65.6	65.5
**3**	45.8	45.9	46.7	46.6	45.3	45.4
**3a**	68.9	69.6	71.5	72.0	70.7	70.8
**4**	47.9	47.9	42.8	43.2	^b^	^b^
**5**	116.8	117.4	148.7	148.7	117.3	117.4
**6**	145.2	146.0	138.9	138.9	145.3	146.0
**7**	146.5	147.4	147.6	147.6	147.4	147.3
**8**	112.1	112.5	107.9	107.8	112.4	112.5
**9**	128.6	128.9	130.2	130.2	129.2	129.2
**10**	133.9	134.0	126.4	126.2	133.7	133.8
**1'**	137.1	138.8	139.4	139.5	138.0	138.8
**2'**	107.8	114.4	107.0	107.1	108.0	114.0
**3'**	148.6	148.7	149.0	149.0	149.3	149.0
**4'**	135.1	145.0	134.5	134.6	133.7	145.3
**5'**	148.6	116.1	149.0	149.0	149.3	116.0
**6'**	107.8	123.2	107.0	107.1	108.0	123.5
**1''**	105.1	105.2	104.8	104.3	103.9	103.9
**2''**	75.1	75.2	75.2	75.1	75.1	75.0
**3''**	77.4	77.9	77.9	78.0	78.0	77.9
**4''**	71.8	71.7	71.7	71.6	71.5	71.5
**5''**	78.0	78.2	78.2	78.2	78.2	78.3
**6''**	63.0	62.5	62.8	62.7	62.5	62.5
**5-OCH_3_**	-	-	60.2	60.1	-	-
**7-OCH_3_**	56.1	56.4	56.6	56.6	56.4	56.3
**3'-OCH_3_**	56.7	56.5	56.9	56.9	56.9	56.6
**5'-OCH_3_**	56.7	-	56.9	56.9	56.9	-

* Acetone-*d*_6_; ^b^ Signal hidden under MeOD.

## 3. Experimental

### 3.1. General

Melting points were measured without correction on a binocular microscopic X-5 melting point apparatus (Beijing, China). FTIR data were recorded on a PerkinElmer spectrophotometer (Spetrum One) equipped with a DGTS detector. ^1^H and ^13^C-NMR data were obtained at 600 MHz and 150 MHz, respectively, in MeOD, C_5_D_5_N and acetone-*d*_6_, on a Bruker Av 600 instrument. Chemical shifts are expressed in δ (ppm) with TMS as internal standard. P-HPLC was run on a Shimadzu LC-8A equipped with a SPD-10A VP detector and an AQ-C18 column (20 × 250 mm, 10 μm). Open column chromatography (CC) was carried out using silica gel (200–300 mesh, Qingdao Marine Chemical Ltd., Qingdao, China), RP-18 (ODS-AQ-HG, YMG*GEL, 5.0 × 60 cm, 12 nm, S-50 μm, Lot: 9955), and Sephadex LH-20 (2.0 × 60 cm, 20–100 μm, GE Healthcare, Uppsala, Sweden). TLC was performed on silica gel plates (Qingdao Marine Chemical Ltd.). Except for methanol (MeOH) which was of chromatographic grade, the other reagents used were analytical grade and purchased from the Tanjin Damao Chemical Reagent Factory (Tanjin, China).

### 3.2. Plant Materials

The roots of *Averrhoa carambola* L. were collected from Linshan County, Guangxi Province, China, in June 2010 and were identified by Prof. Maoxiang Lai. The voucher specimen (No. 20100605) was deposited in the herbarium of the Guangxi Institute of Chinese Medicine & Pharmaceutical Science (Guangxi, China).

### 3.3. Extraction and Isolation

The powder of air-dried roots of *Averrhoa carambola* L. (12 kg) was extracted three times with 60% aq. EtOH under reflux (96 L, 1 h each time). The ethanolic solution was concentrated under vacuum to yield a syrup-like extract which was suspended in H_2_O and then extracted with cyclohexane (3 × 20 L), EtOAc (3 × 20 L) and *n*-BuOH (3 × 20 L), respectively. The *n*-BuOH extract (153 g) was subjected to silica gel open CC (13 × 100 cm, 200–300 mesh, 1.5 kg), eluting successively with a chloroform/MeOH gradient (100:0 to 0:100) to afford 13 fractions Fr.1–Fr.13.

Fr.7 (9.4 g) was subjected to silica gel CC (6 × 120 cm, 200–300 mesh, 100 g), eluting successively with a petroleum ether/EtOAc gradient (100:0, 2:1, 1:1, 0:100, each 1.0 L) and EtOAc/MeOH (15:0, 10:1, 5:1, 4:1, 3:1, 1:1, 0:100, each 1.0 L) to obtain seven subfractions Fr.71–Fr.77. Then Fr.72 (1.1 g), Fr.73 (3.2 g) was subjected to Sephadex LH-20 CC with MeOH and gave sub-fractions Fr.721–Fr.723, Fr.731–Fr.733, respectively. Fr.732 was further separated on a RP-18 CC, eluting with a MeOH/H_2_O gradient (10%, 30%, 50%, 70%, 100%, each 150 mL), and five fractions (Fr.7321–7325) were obtained from it. Compound **1** (0.8 mg, t_R_ = 69.0 min), and compound **2** (3.2 mg, t_R_ = 74.0 min) were isolated by P-HPLC (MeOH/H_2_O 25:75, 8 mL/min, 203 nm) from Fr.7321.

Fr.8 (16.6 g) was subjected to silica gel CC (6 × 120 cm, 200–300 mesh, 160 g), eluting successively with gradient petroleum ether/EtOAc (100:0, 1:1, 0:100) and EtOAc/MeOH (18:0, 10:1, 8:1, 6:1, 4:1, 2:1, 0:100) to obtain six sub-fractions Fr.81–Fr.86. Compounds **3** (70.3 mg) and **4** (12.8 mg) were obtained from Fr.84. Then Fr.83, Fr.84 were further purified successively by Sephadex LH-20 CC with MeOH, and afforded fractions Fr.831–Fr.833, Fr.841–Fr.843. Fr.831, Fr.842 were purified by RP-18 CC and eluted with MeOH/H_2_O gradient (10%, 20%, 30%, 50%, 100%, each 150 mL). Ten fractions Fr.8311–Fr.8315, Fr.8421–8425 were obtained from Fr.831, Fr.842, respectively. After that, Fr.8311 was subjected to silica gel CC (1.5 × 120 cm, 200–300 mesh, 30 g), eluting successively with a dichloromethane/MeOH gradient (100:0, 20:1, 18:1, 15:1, 12:1, 9:1, 6:1, 3:1, 1:1, 0:100, each 450 mL) to give eight sub-fractions Fr.83111–Fr.83118. Fr.83113 was further purified successively by P-HPLC (MeOH/H_2_O 25:75, 8.0 mL/min, 203 nm) to produce compound **5** (4.2 mg, t_R_ = 13.0 min). Fr.8421 was isolated by silica gel CC (1.5 × 120 cm, 200–300 mesh, 60 g) with dichloromethane/MeOH gradient (100:0, 20:1, 18:1, 16:1, 10:1, 0:100, each 200 mL) and monitored by TLC to give eight fractions Fr.84211–84218. Fr.8422 was followed by RP-18 CC (5.0 × 60 cm) and eluted with a MeOH/H_2_O gradient (10%, 20%, 30%, 50%, 100%, each 150 mL) to yield five fractions Fr.84221–Fr.84225. Then Fr.84215, Fr.8423 were purified in turns by P-HPLC (MeOH/H_2_O 28:72, 8.0 mL/min, 203 nm) to give compound **6** (418.9 mg, t_R_ = 41.0 min), compound **7** (534.7 mg, t_R_ = 48.0 min), compound **8** (2.5 mg, t_R_ = 81.5 min), and compound **9** (1.0 mg, t_R_ = 95.5 min).

Fr.9 (12.7 g) was subjected to silica gel CC (6 × 120 cm, 200–300 mesh, 130 g) and eluted successively with a EtOAc/MeOH gradient (100:0, 15:1, 10:1, 8:1, 5:1, 4:1, 3:1, 2:1, 1:1, 0:100, each 1.0 L), to give six sub-fractions Fr.91–Fr.96. Then Fr.92 (7.8 g) was separated by Sephadex LH-20 CC with MeOH and produced five fractions Fr.921–Fr.925. Fr.923 (4.8 g) was purified by RP-18 CC, eluting with a MeOH/H_2_O gradient (10%, 20%, 30%, 50%, 100%, each 150 mL) to obtain six fractions Fr.9231–Fr.9236. Then Fr.9232 was further purified by P-HPLC (MeOH/H_2_O 25:75, 8.0 mL/min, 203 nm) to give compound **10** (3.2 mg, t_R_ = 12.0 min), and compound **11** (14.5 mg, t_R_ = 27.0 min). Fr.9231 was isolated by silica gel CC (1.5 × 120 cm, 200–300 mesh, 3.5 g) with a dichloromethane/MeOH gradient (100:0, 20:1, 16:1, 12:1, 10:1, 7:1, each 200 mL) to afford 12 fractions Fr.92311–Fr.92322. Fr.92318, Fr.92319, Fr.92321 were further purified successively by P-HPLC (8.0 mL/min, 203 nm) with MeOH/H_2_O (16:84, 20:80, 13:87), to produce compound **12** (1.4 mg, t_R_ = 10.0 min), compound **13**(17.2 mg, t_R_ = 17.0 min), compound **14** (10.3 mg, t_R_ = 21.0 min), compound **15** (7.6 mg, t_R_ = 23.0 min).

*3 ,4,5- Trimethoxyphenol-1-O- β -D- glucopyranoside* (**1**). White needles, m.p. 201–203 °C, 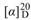
−28.0 (*c* 0.3, MeOH), IR (KBr) cm^−1^: 3274.0, 2924.4, 1602.8, 1507.8, 1466.4, 1419.2, 1384.7, 1228.3, 1197.1, 1166.7, 1127.9, 1074.9, 1055.5, 1036.7, 1019.1, 998.9, 835.0, 819.7, 781.4. ^1^H-NMR (MeOD) δ: 3.42–3.47 (4H, m, overlaped, H-2', 3', 4', 5'), 3.66 (1H, dd, *J* = 12.1, 6.7 Hz, H-6'a), 3.70 (3H, s, 4-OMe), 3.81 (6H, s, 3, 5-OMe), 3.92 (1H, dd, *J* = 12.1, 2.4 Hz, H-6'b), 4.82 (1H, d, *J* = 7.3 Hz, H-1'), 6.49 (2H, s, H-2, 6) [11].

*Benzyl-1-O- β -D- glucopyranoside* (**2**). White needles, m.p. 212.3–213.7 °C, 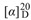
−41.0 (*c* 0.2, MeOH), IR (KBr) cm^−1^: 3273.6, 2937.6, 1604.1, 1507.8, 1466.5, 1228.6, 1196.9, 1128.4, 1074.9, 1018.4, 999.3, 669.1. ^1^H-NMR (MeOD) δ: 7.42 (2H, d, *J* = 7.4 Hz, H-2, 6), 7.26–7.34 (3H, m, overlapped, H-3, 4, 5), 4.93 (1H, d, *J* = 11.8 Hz, H-7a), 4.68 (1H, d, *J* = 11.8 Hz, H-7b), 4.36 (1H, d, *J* = 7.7 Hz, H-1'), 3.90 (1H, dd, *J* = 2.2, 12.0 Hz, H-6'a), 3.68 (1H, dd, *J* = 5.6, 12.0 Hz, H-6'b) [12].

*(+)-5'-Methoxyisolariciresinol 3α-O-β -D- glucopyranoside* (**3**). White powder, m.p. 163.5–165.0 °C, 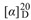
+11.0 (*c* 0.1, MeOH), IR (KBr) cm^−1^: 3459.6, 2901.8, 1591.6, 1513.6, 1464.4, 1321.4, 1280.3, 1226.1, 1120.0, 1020.7, 949.3, 930.9. ^1^H-NMR (acetone-*d*_6_) δ: 7.18 (1H, s, H-8), 7.06 (1H, s, H-5), 6.54 (2H, s, H-2', 6'), 4.69 (1H, d, *J* = 3.7 Hz, H-1''), 4.25 (1H, d, *J* = 3.7 Hz, H-3a), 4.18 (1H, d, *J* = 4.2 Hz, H-3b), 4.09 (1H, dd, *J* = 10.0, 2.5 Hz, H-1b), 3.79 (3H, s, OMe), 3.77 (6H, s, 3', 5'-OMe), 3.61–3.72 (4H, m, overlaped, H-2'', 3'', 4'', 5''), 3.57–3.55 (1H, t), 3.41–3.37 (1H, m), 3.33 (1H, dd, *J* = 9.4, 4.1 Hz, H-6a), 3.26–3.25 (3H, m), 3.15 (1H, dd, *J* = 10.0, 2.9 Hz, H-1a), 2.79 (1H, dd, *J* = 16.1, 4.8 Hz, H-6b), 2.08 (1H, m, H-3) [13].

*(+)-Isolariciresinol 3α-O-β -D- glucopyranoside* (**4**). White powder, m.p. 146.0–148.0 °C, 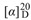
+15.2 (*c* 0.32, MeOH), IR (KBr) cm^−1^: 3901.8, 3478.7, 1698.7, 1616.3, 1541.4, 1512.5, 1502.5, 1456.7, 1275.6, 1025.9. ^1^H-NMR (MeOD) δ: 6.79 (1H, d, *J* = 2.0 Hz, H-2'), 6.74 (1H, d, *J* = 8.0 Hz, H-5'), 6.65 (1H, br.s, H-8), 6.64 (1H, dd, *J* = 8.0, 2.0 Hz, H-6'), 4.12 (1H, d, H-3a), 4.07 (1H, t, H-3b), 3.81 (3H, s, OMe), 3.80 (3H, s, OMe), 3.77–3.70 (2H, m), 3.29–3.20 (4H, m, partly overlapped), 2.86–2.78 (2H, m, H-1a, H-1b), 2.10–2.07 (1H, m, H-3), 1.85–1.88 (1H, m, H-2) [13].

*Koaburaside* (**5**). White powder, m.p. 238.0 °C, 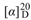
−21.0 (*c* 0.25, MeOH), IR (KBr) cm^−1^: 3704.9, 2925.1, 1736.5, 1601.7, 1508.3, 1484.4, 1434.6, 1222.4, 1126.2, 1073.6, 996.1, 818.4, 639.0. ^1^H-NMR (MeOD) δ: 6.13 (2H, s, H-2, 6), 3.79 (6H, 2 × OMe), 4.66 (1H, d, *J* = 7.4 Hz, H-1'), 3.44 (1H, dd, *J* = 9.7, 2.3 Hz, H-2'), 3.40 (2H, dd, overlapped, H-3', 4'), 3.50-3.59 (1H, m, H-5'), 3.75 (1H, br. d, *J* = 17.3, 8.76 Hz, H-6'a), 3.67 (1H, dd, *J* = 11.8, 5.2 Hz, H-6'b) [14].

*(+)-Lyoniresinol 3 α -O- β -D- glucopyranoside* (**6**). Light yellow powder, m.p. 119–120°C. 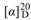
+21.0 (*c* 0.4, MeOH), IR (KBr) cm^−1^: 3365.9, 2936.9, 1614.7, 1517.1, 1500.7, 1457.2, 1426.5, 1322.9, 1218.7, 1111.9, 900.8, 807.0, 632.7. ^1^H-NMR (C_5_D_5_N) δ: 6.73 (1H, s, H-8), 7.05 (2H, s, H-2', H-6'), 5.14 (1H, d, *J* = 6.1 Hz, H-4), 4.97 (1H, partly overlapped, H-1''), 4.52 (1H, dd, *J* = 11.7, 2.5 Hz, H_A_-2a or H_A_-3a), 4.42 (1H, dd, *J* = 10.0, 4.3 Hz, H_B_-2b or H_B_-3b), 4.36 (1H, d, *J* = 11.8, 5.3 Hz, H_A_-2a or H_A_-3a), 4.26–4.22 (2H, m, H-6''), 4.14–3.94 (4H, m, overlapped, H-2'',3'',4'',5''), 3.77 (3H, s, 7-OMe), 3.75 (3H, s, 5-OMe), 3.71 (6H, s,3, 5'-OMe), 3.14 (1H, dd, *J* = 14.7, 12.0 Hz, H-1a), 3.07 (1H, dd, *J* = 15.2, 4.4 Hz, H-1b ), 2.73 (1H, m, H-3), 2.14 (1H, m, H-2) [13,15].

*(−)-Lyoniresinol 3 α -O- β -D- glucopyranoside* (**7**). Light yellow powder, m.p. 133.0–134.5 °C, 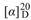
−45.5 (*c* 0.3, MeOH), IR (KBr) cm^−1^: 3390.3, 2936.0, 1614.2, 1517.3, 1501.2, 1461.3, 1426.7, 1323.9, 1218.5, 1111.9, 901.0, 806.1, 626.2. ^1^H-NMR (MeOD) δ: 6.57 (1H, s, H-8), 6.41 (2H, s, H-2', H-6'), 4.22 (1H, d, *J* = 6.5 Hz, H-4), 4.13 (1H, d, *J* = 7.8 Hz, H-1"), 3.87–3.83 (5H, m), 3.85 (3H, s, OMe), 3.75 (6H, s, 2 × OMe), 3.69 (1H, dd, *J* = 11.9, 5.5 Hz, H-6"a). 3.63–3.57 (3H, m), 3.32–3.27 (m, mostly overlapped), 3.33 (3H, s, OMe), 3.22–3.14 (2H, m), 2.71–2.64 (2H, m, H-1a, H-1b), 2.15–2.11 (1H, m, H-3), 1.71–1.65 (1H, m, H-2) [13].

*(−)-5'-Methoxyisolariciresinol 3α-O-β -D- glucopyranoside* (**8**). Light yellow powder, m.p. 157.0–158.0 °C, 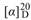
−40.0 (*c* 0.5, MeOH), IR (KBr) cm^−1^: 3420.8, 2927.5, 1611.7, 1512.8, 1456.9, 1384.5, 1219.2, 1117.4. ^1^H-NMR (MeOD) δ: 6.68 (1H, s, H-8), 6.47 (2H, s, H-2', H-6'), 6.24 (1H, s, H-5), 4.08 (1H, d, *J* = 7.8 Hz, H-1"), 3.79–3.66 (6H, m), 3.83 (3H, s, OMe), 3.82 (6H, s, 2×OMe), 3.32–3.29 (2H, m), 3.19 (1H, dd, *J* = 8.9, 8.0 Hz), 3.08–3.06 (1H, m), 2.91 (1H, dd, *J* = 15.8, 10.3 Hz, H-1a), 2.77 (1H, dd, *J* = 16.0, 3.6 Hz, H-1b), 2.02–1.98 (2H, m, H-2, H-3) [13].

*(−)-Isolariciresinol 3 α -O- β -D- glucopyranoside* (**9**). Light yellow powder, m.p. 209–210°C, 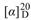
−32.5 (*c* 0.1, MeOH), IR (KBr) cm^−1^: 3828.9, 3710.8, 2924.5, 2853.2, 1844.5, 1698.4, 1594.3, 1555.0, 1541.4, 1512.6, 1464.3, 1456.8, 1384.3, 1270.9, 1125.6, 1075.2, 1033.4. ^1^H-NMR (MeOD) δ: 6.74 (1H, d, *J* = 8.0 Hz, H-5'), 6.69 (1H, d, *J* = 1.9 Hz, H-2'), 6.65 (1H, br. s, H-8), 6.64 (1H, d, *J* = 2.0 Hz, H-6'), 6.19 (1H, s, H-5), 4.04 (1H, d, *J* = 7.8 Hz, H-1"). 3.85–3.63 (m), 3.81 (3H, s, OMe), 3.80 (3H, s, OMe), 3.35–3.25 (m, partly overlapped), 3.16 (1H, m), 3.06–3.04 (1H, m), 2.89–2.86 (1H, m, H-1a), 2.75 (1H, br. dd, *J* = 15.4, 4.0 Hz, H-1b), 1.97 (2H, m, H-2, H-3) [13].

*3,5-Dimethoxy-4-hydroxyphenyl 1-O-β -apiofuranosyl (1''→6')-O-β -D- glucopyranoside* (**10**). Light yellow powder, m.p. 126.5–128.0 °C, 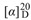
−42.0 (*c* 0.6, MeOH), IR (KBr) cm^−1^: 3750.9, 3725.7, 3627.5, 3564.8, 2924.0, 1602.6, 1507.8, 1486.8, 1455.8, 1384.1, 1222.6, 1124.4, 1043.4, 818.3. ^1^H-NMR (MeOD) δ: 6.13 (2H, s, H-2, 6), 3.80 (6H, s, 3, 5-OMe), 4.24 (1H, d, *J* = 7.4 Hz, H-1'), 3.47–3.38 (6H, m, overlapped), 3.82 (1H, dd, *J* = 11.5, 5.3 Hz, H-6'a), 4.00 (1H, dd, *J* = 11.5, 2.1 Hz, H-6'b), 4.65 (1H, d, *J* = 7.7 Hz, H-1''), 3.76–3.72 (2H, m, overlapped) [16].

*3,4,5-Trimethoxyphenyl 1-O-β-apiofuranosyl (1 ''→6')- β-glucopyranoside* (**11**). White needles, m.p. 203.0–205.0 °C, 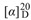
−62.0 (*c* 0.8, MeOH), IR (KBr) cm^−1^: 3748.6, 3335.5, 1698.9, 1616.8, 1541.3, 1512.0, 1473.0, 1052.7. ^1^H-NMR (MeOD) δ: 6.47 (2H, s, H-2, 6), 3.82 (6H, s, 3, 5-OMe), 3.71 (3H, s, 4-OMe), 4.28 (1H, d, *J* = 7.6 Hz, H-1'), 4.82 (1H, d, *J* = 7.6 Hz, H-1''), 3.84 (2H, dd, *J* = 11.4, 5.3 Hz, H-4'') [17].

*Methoxyhydroquinone-4- β -D- glucopyranoside* (**12**). White powder, m.p. 211.0–213.0 °C, 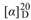
−34.5 (*c* 0.15, MeOH), IR (KBr) cm^−1^: 3748.9, 3728.4, 2923.8, 1716.0, 1576.1, 1541.3, 1513.0, 1456.7, 1383.1, 1296.7, 1244.4, 1223.7, 1197.3, 1169.4, 1082.3, 1045.7, 989.4, 942.4, 840.1, 804.6. ^1^H-NMR (MeOD) δ: 6.80 (1H, d, *J* = 2.7 Hz, H-5), 6.69 (1H, d, *J* = 8.6 Hz, H-2), 6.59 (1H, dd, *J* = 8.6, 2.7 Hz, H-6), 3.83 (3H, s, OMe), 4.74 (1H, d, *J* = 7.4 Hz, H-1'), 3.46–3.35 (4H, overlapped), 3.90 (1H, dd, *J* = 12.0, 2.2 Hz, H-6'a), 3.69 (1H, dd, *J* = 12.0, 5.8 Hz, H-6'b) [18,19].

*(2S)-2-O- β -D- Glucopyranosyl-2-hydroxyphenylacetic acid* (**13**). Amorphous powder, m.p. 117.0–119.0 °C, 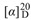
−87.5 (*c* 0.4, MeOH), IR (KBr) cm^−1^: 3748.7, 3628.3, 3176.7, 2883.1, 1676.1, 1590.1, 1555.3, 1541.6, 1512.0, 1497.0, 1456.0, 1411.5, 1308.1, 1196.4, 1156.2, 1076.0, 897.1, 766.7, 702.8. ^1^H-NMR (MeOD) δ: 7.52 (2H, t, H-2, 6), 7.37–7.32 (3H, m, H-3, 4, 5), 5.29 (1H, s, H-7), 4.51 (1H, d, *J* = 7.8 Hz, H-1'), 3.87 (1H, d, *J* = 11.8 Hz, H-6'a), 3.67 (1H, dd, *J* = 12.0, 5.0 Hz, H-6'b), 3.46–3.40 (1H, m), 3.35 (1H, d, *J* = 8.0 Hz) [20,21].

*3-Hydroxy-4-methoxyphenol 1-O- β -D- apiofuranosyl-(1''→6')-O- β -D- glucopyranoside* (**14**). White powder, m.p. 226.0–228.0 °C, 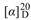
−47.5 (*c* 0.3, MeOH), IR (KBr) cm^−1^: 3391.5, 1618.4, 1513.9, 1384.6, 1226.6, 1201.5, 1114.3, 1070.9, 856.2. ^1^H-NMR (MeOD) δ: 6.76 (1H, d, *J* = 9.0 Hz, H-5), 6.71 (1H, d, *J* = 2.4 Hz, H-2), 6.62 (1H, dd, *J* = 8.4, 3.0 Hz, H-6), 3.84 (3H, s, 4-OMe), 4.31 (1H, d, *J* = 7.2 Hz, H-1'), 3.61–3.58 (1H, m), 3.48–3.44 (1H, m), 3.79 (1H, dd, *J* = 11.4, 6.0 Hz, H-6'a), 4.10 (1H, dd, *J* = 11.4, 1.8 Hz, H-6'b), 4.74 (1H, d, *J* = 7.2 Hz, H-1''), 3.42–3.37 (3H, m, overlapped), 3.21–3.14 (3H, m, overlapped) [22].

*4-Hydroxy-3-methoxyphenol 1-O- β -D- apiofuranosyl-(1 ''*→*6 ' )-O- β -D- glucopyranoside* (**15**). Light yellow powder, m.p. 124.0–125.0 °C, 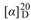
−54.0 (*c* 0.3, MeOH), IR (KBr) cm^−1^: 3750.8, 3725.8, 3654.8, 3627.8, 2926.8, 1675.1, 1605.6, 1541.5, 1512.5, 1456.6, 1384.7, 1301.8, 1214.3, 1164.0, 1063.5, 954.2, 834.0, 807.0. ^1^H-NMR (MeOD) δ: 7.00 (1H, d, *J* = 8.7 Hz, H-2), 6.47 (1H, d, *J* = 2.8 Hz, H-5), 6.32 (1H, dd, *J* = 8.5, 2.8 Hz, H-6), 3.81 (3H, s, 3-OMe), 4.66 (1H, d, *J* = 7.5 Hz, H-1'), 3.43–3.42 (2H, m, overlapped), 3.48–3.45 (1H, m, overlapped), 3.62 (1H, dd, *J* = 11.1, 6.4 Hz, H-6'a), 3.98 (1H, dd, *J* = 11.1, 1.9 Hz, H-6'b), 4.98 (1H, d, *J* = 2.3 Hz, H-1''), 3.89 (1H, d, *J* = 2.3 Hz, H-2''), 3.74 (1H, d, *J* = 9.6 Hz, H-4''a), 3.93 (1H, d, *J* = 9.7 Hz, H-4''b), 3.58 (2H, s) [16].

## 4. Conclusions

The ^1^H and ^13^C-NMR data of 15 compounds isolated from the roots of *Averrhoa carambola* L.for the first time were identical with the literature values.

## Supplementary Materials

FTIR, ^1^H and ^13^C-NMR spectrum of compounds **1**–**15** are available as Supplementary Materials which can be accessed at: http://www.mdpi.com/1420-3049/17/10/12330/s1.
