# Supplementary material for: Phenolic and Lignan Glycosides from the Butanol Extract of Averrhoa carambola L. Root
Source: Molecules. 2012 Oct 19;17(10):12330–40. doi: 10.3390/molecules171012330 (PMC6268650; doi:10.3390/molecules171012330)

Supporting Information

List of contents

Figure 1a ^{1}H-NMR spectrum (MeOD, 600 MHz) of compound **1**.
Figure 1b ^{13}C-NMR spectrum (MeOD, 150 MHz) of compound **1**.
Figure 2a ^{1}H-NMR spectrum (MeOD, 600 MHz) of compound **2**.
Figure 2b ^{13}C-NMR spectrum (MeOD, 150 MHz) of compound **2**.
Figure 3a ^{1}H-NMR spectrum (Acetone-d6, 600 MHz) of compound **3**.
Figure 3b ^{13}C-NMR spectrum (Acetone-d6, 150 MHz) of compound **3**.
Figure 4a ^{1}H-NMR spectrum (MeOD, 600 MHz) of compound **4**.
Figure 4b ^{13}C-NMR spectrum (MeOD, 150 MHz) of compound **4**.
Figure 5a ^{1}H-NMR spectrum (MeOD, 600 MHz) of compound **5**.
Figure 5b ^{13}C-NMR spectrum (MeOD, 150 MHz) of compound **5**.
Figure 6a ^{1}H-NMR spectrum (C_5D_5N, 600 MHz) of compound **6**.
Figure 6b ^{13}C-NMR spectrum (MeOD, 150 MHz) of compound **6**.
Figure 7a ^{1}H-NMR spectrum (MeOD, 600 MHz) of compound **7**.
Figure 7b ^{13}C-NMR spectrum (MeOD, 150 MHz) of compound **7**.
Figure 8a ^{1}H-NMR spectrum (MeOD, 600 MHz) of compound **8**.
Figure 8b ^{13}C-NMR spectrum (MeOD, 150 MHz) of compound **8**.
Figure 9a ^{1}H-NMR spectrum (MeOD, 600 MHz) of compound **9**.
Figure 9b ^{13}C-NMR spectrum (MeOD, 150 MHz) of compound **9**.
Figure 10a ^{1}H-NMR spectrum (MeOD, 600 MHz) of compound **10**.
Figure 10b ^{13}C-NMR spectrum (MeOD, 150 MHz) of compound **10**.
Figure 11a ^{1}H-NMR spectrum (MeOD, 600 MHz) of compound **11**.
Figure 11b ^{13}C-NMR spectrum (MeOD, 150 MHz) of compound **11**.
Figure 12a ^{1}H-NMR spectrum (MeOD, 600 MHz) of compound **12**.
Figure 12b ^{13}C-NMR spectrum (MeOD, 150 MHz) of compound **12**.
Figure 13a ^{1}H-NMR spectrum (MeOD, 600 MHz) of compound **13**.
Figure 13b ^{13}C-NMR spectrum (MeOD, 150 MHz) of compound **13**.
Figure 14a ^{1}H-NMR spectrum (MeOD, 600 MHz) of compound **14**.
Figure 14b ^{13}C-NMR spectrum (MeOD, 150 MHz) of compound **14**.
Figure 15a ^{1}H-NMR spectrum (MeOD, 600 MHz) of compound **15**.
Figure 15b ^{13}C-NMR spectrum (MeOD, 150 MHz) of compound **15**.
Figure 16 FTIR spectrum (KBr) of compound **1**.
Figure 17 FTIR spectrum (KBr) of compound **2**.
Figure 18 FTIR spectrum (KBr) of compound **3**.
Figure 19 FTIR spectrum (KBr) of compound **4**.
Figure 20 FTIR spectrum (KBr) of compound **5**.
Figure 21 FTIR spectrum (KBr) of compound **6**.
Figure 22 FTIR spectrum (KBr) of compound **7**.
Figure 23 FTIR spectrum (KBr) of compound **8**.
Figure 24 FTIR spectrum (KBr) of compound **9**.
Figure 25 FTIR spectrum (KBr) of compound **10**.

Figure 26 FTIR spectrum (KBr) of compound **11**.
Figure 27 FTIR spectrum (KBr) of compound **12**.
Figure 28 FTIR spectrum (KBr) of compound **13**.
Figure 29 FTIR spectrum (KBr) of compound **14**.
Figure 30 FTIR spectrum (KBr) of compound **15**.

Figure 1a. ^{1}H-NMR spectrum (MeOD, 600 MHz) of compound **1**.

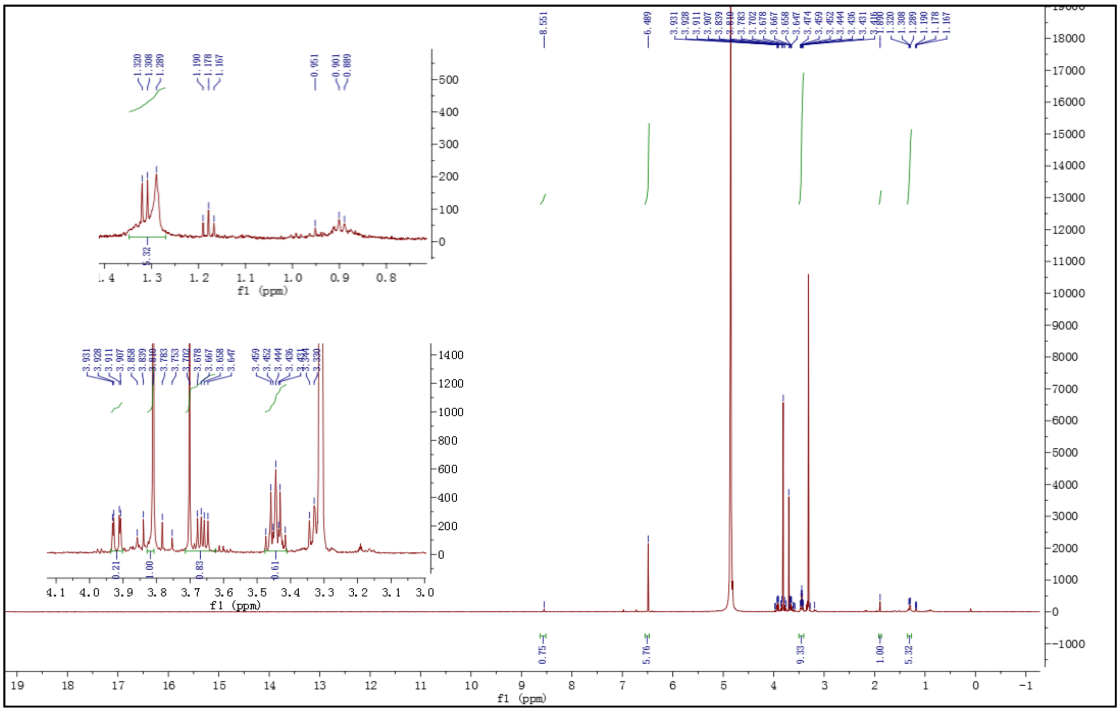

Figure 1b. ^{13}C-NMR spectrum (MeOD, 150 MHz) of compound **1**.

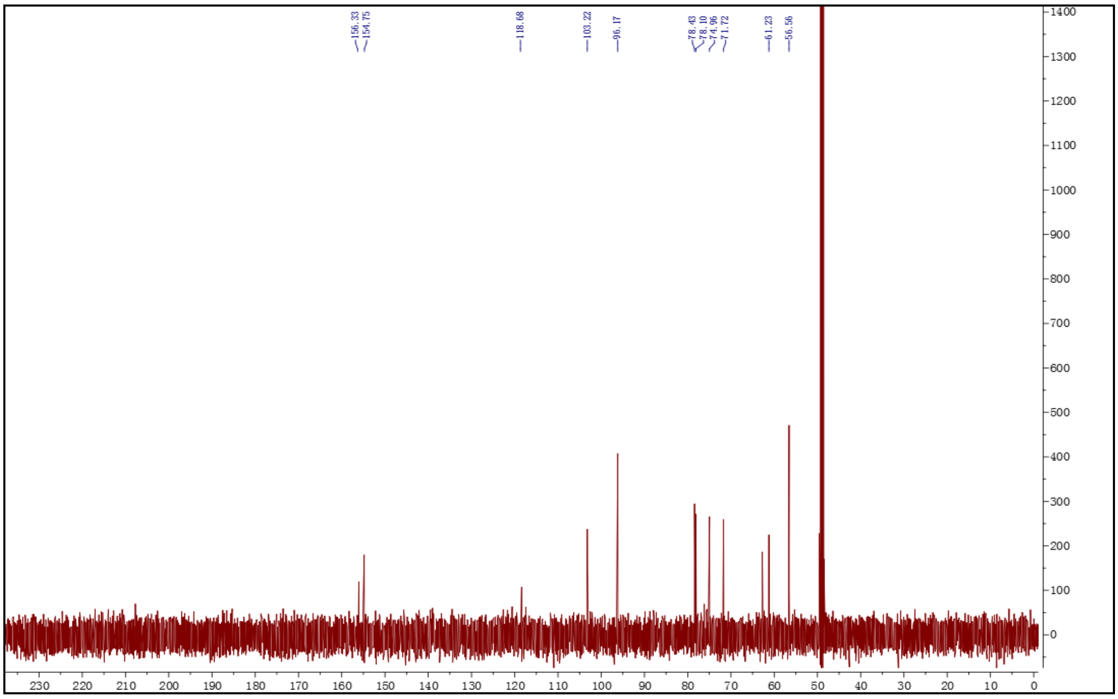

Figure 2a. ^{1}H-NMR spectrum (MeOD, 600 MHz) of compound **2**.

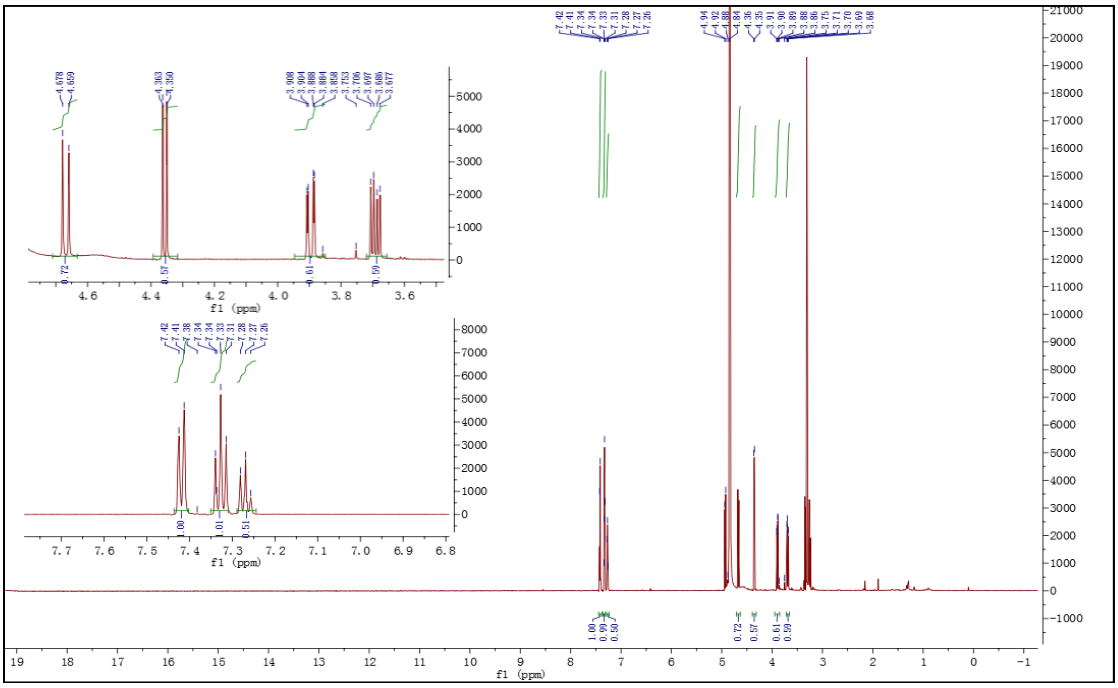

Figure 2b. ^{13}C-NMR spectrum (MeOD, 150 MHz) of compound **2**.

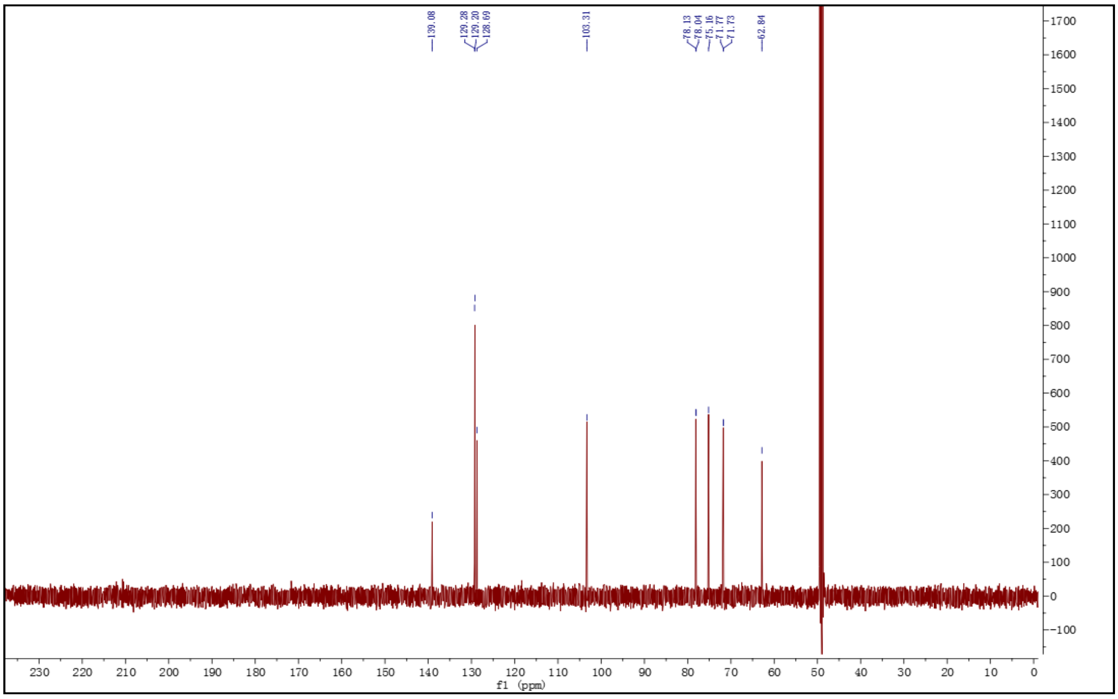

Figure 3a. ^{1}H-NMR spectrum (Acetone-d6, 600 MHz) of compound **3**.

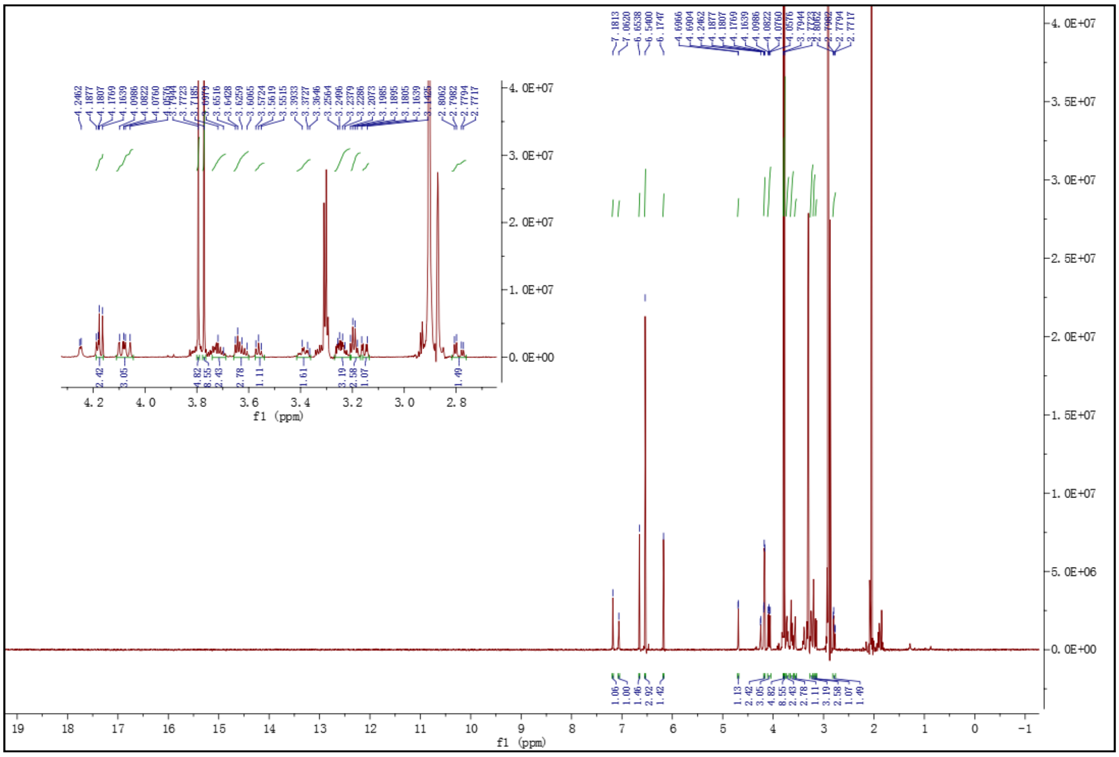

Figure 3b. ^{13}C-NMR spectrum (Acetone-d6, 150 MHz) of compound **3**.

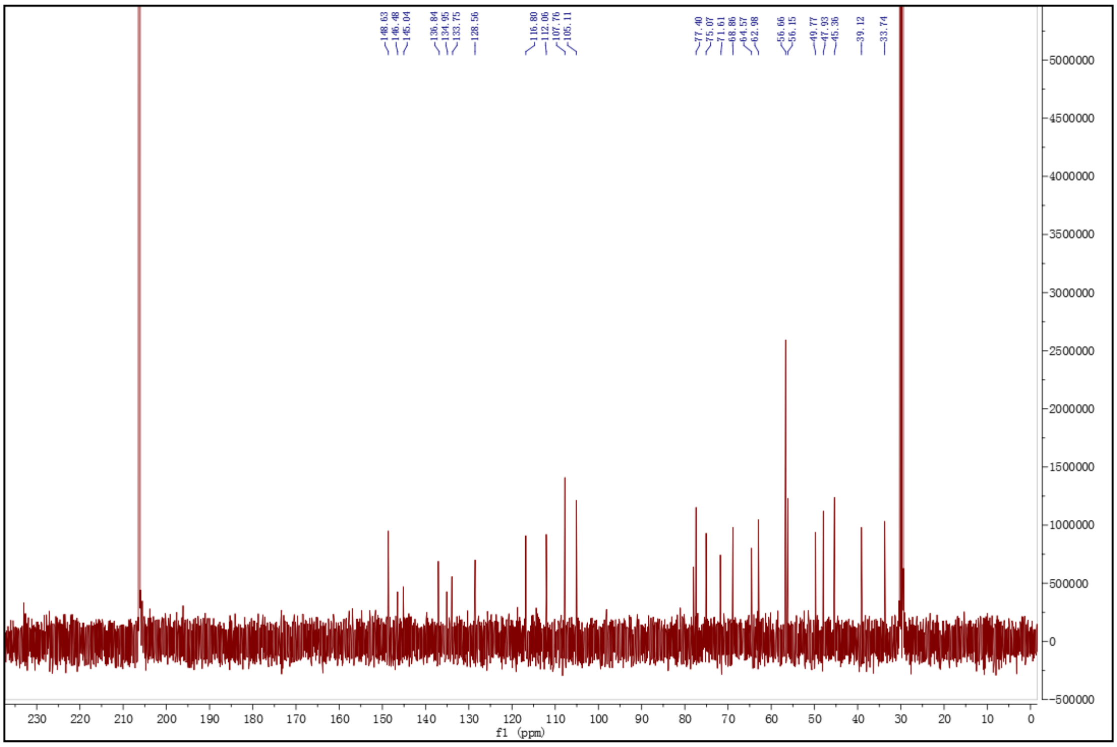

Figure 4a. ^{1}H-NMR spectrum (MeOD, 600 MHz) of compound **4**.

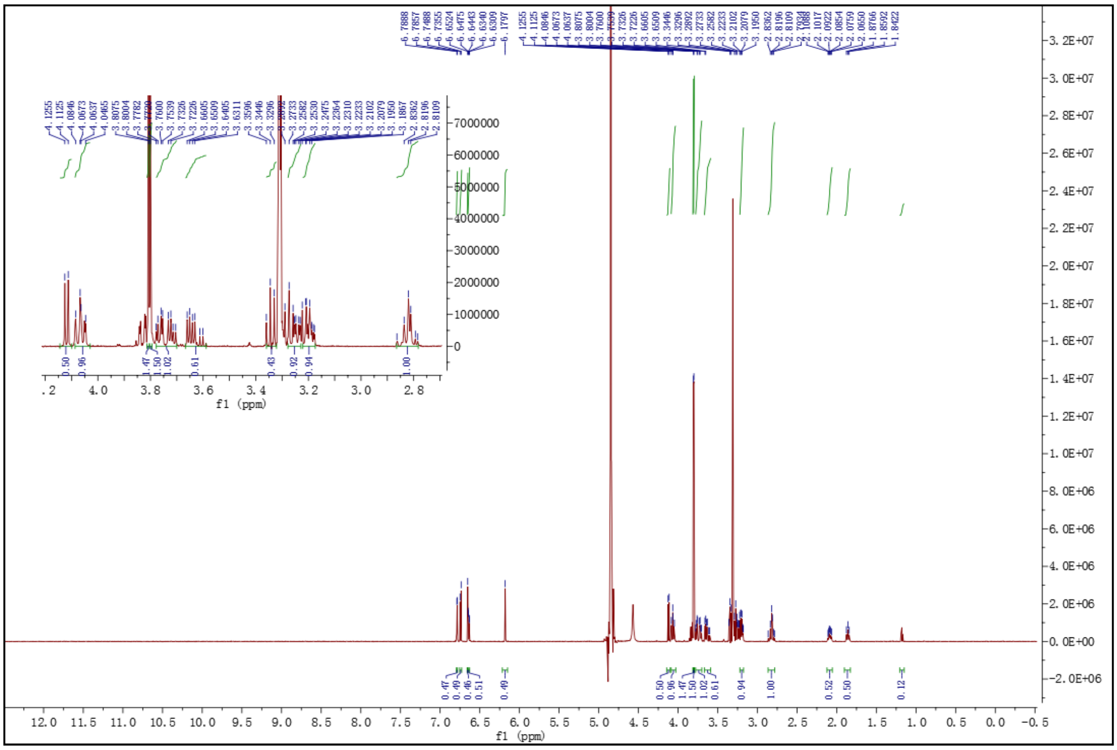

Figure 4b. ^{13}C-NMR spectrum (MeOD, 150 MHz) of compound **4**.

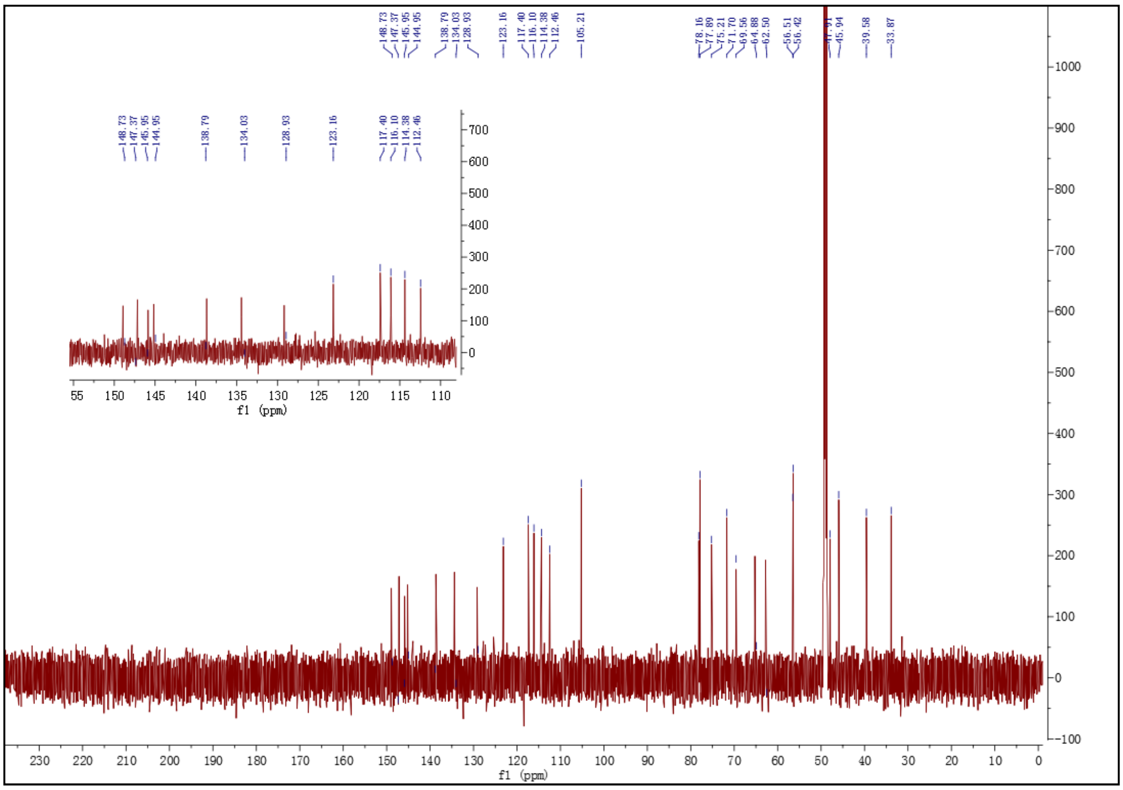

Figure 5a. ^{1}H-NMR spectrum (MeOD, 600 MHz) of compound **5**.

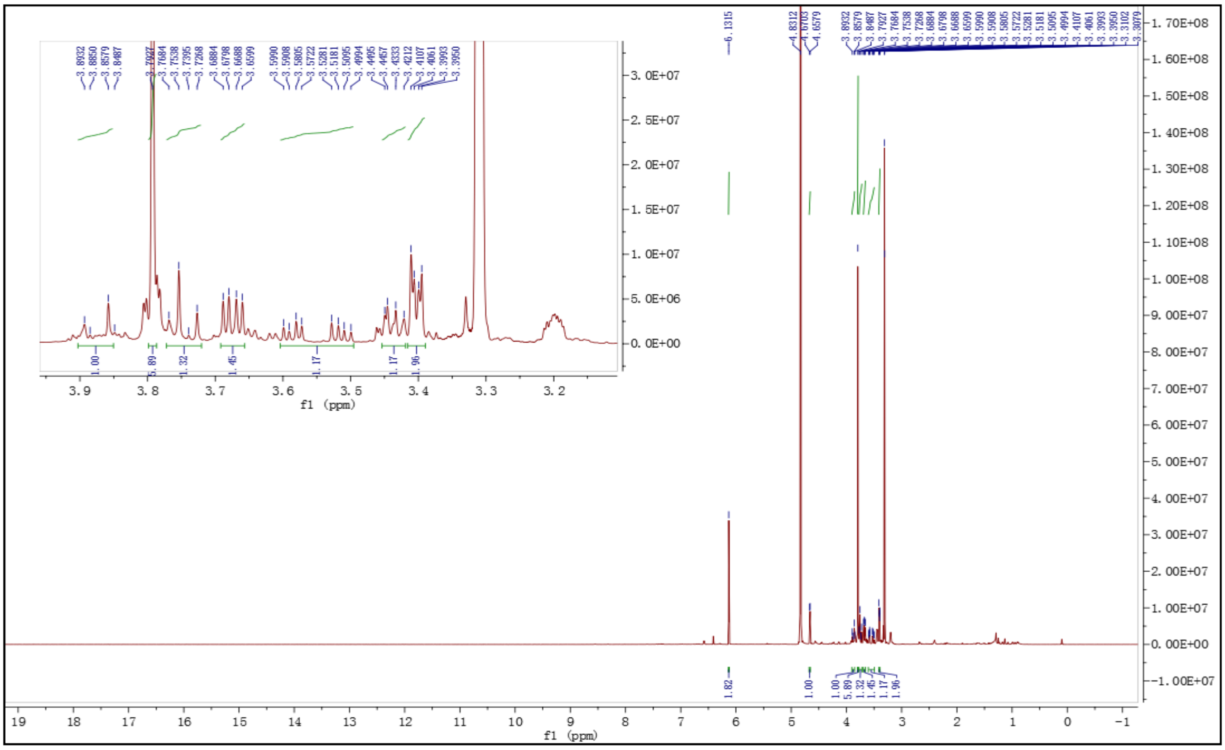

Figure 5b. ^{13}C-NMR spectrum (MeOD, 150 MHz) of compound **5**.

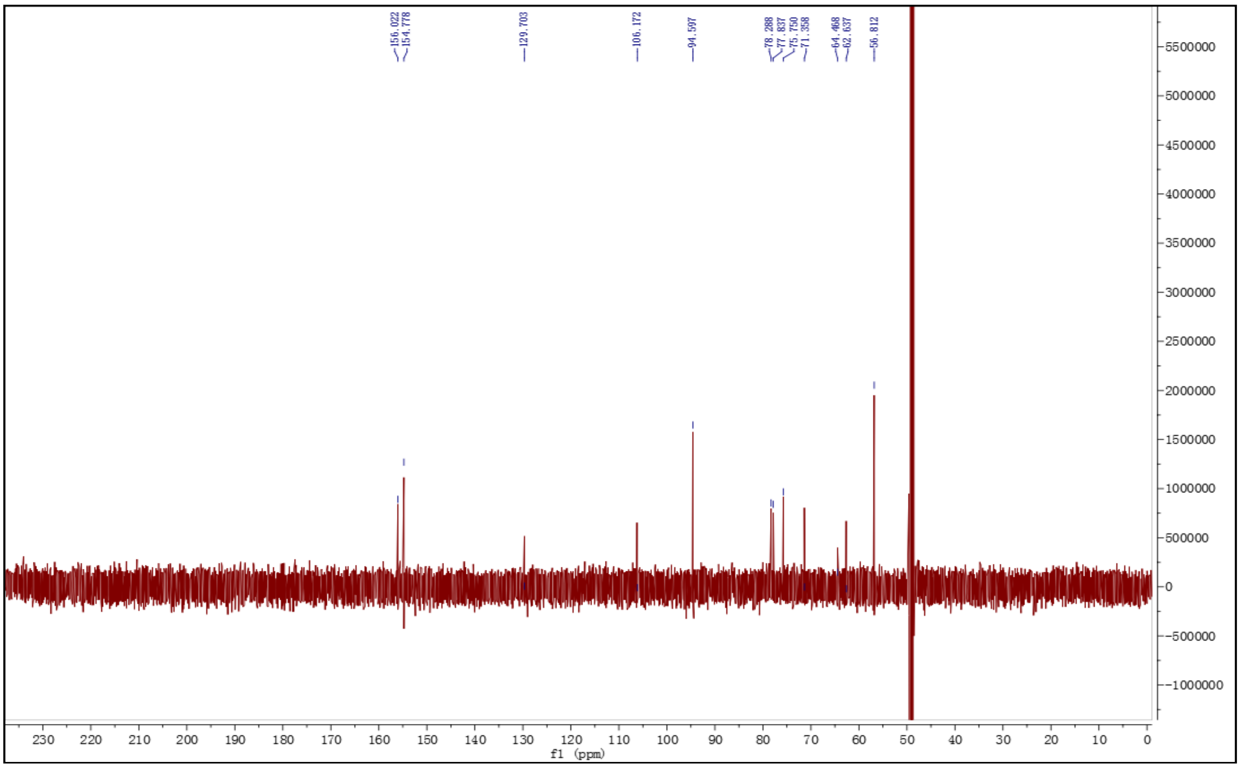

Figure 6a. ^{1}H-NMR spectrum (C_5D_5N, 600 MHz) of compound **6**.

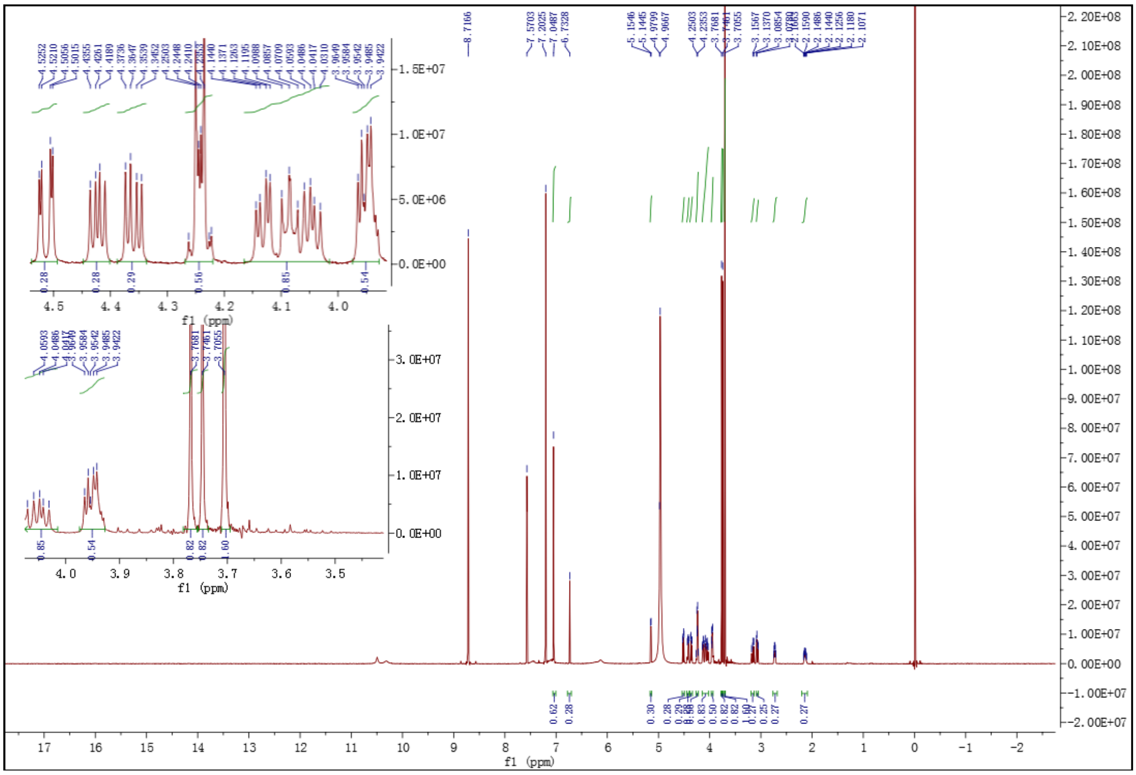

Figure 6b. ^{13}C-NMR spectrum (MeOD, 150 MHz) of compound **6**.

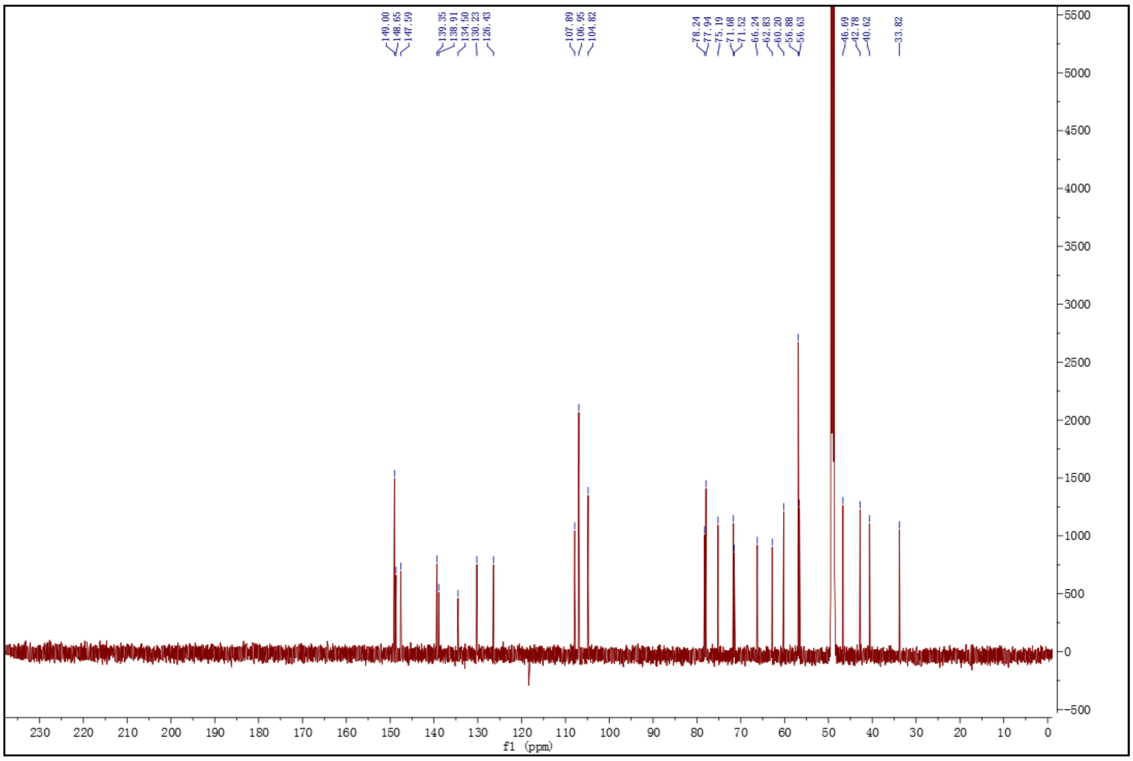

Figure 7a. ^{1}H-NMR spectrum (MeOD, 600 MHz) of compound **7**.

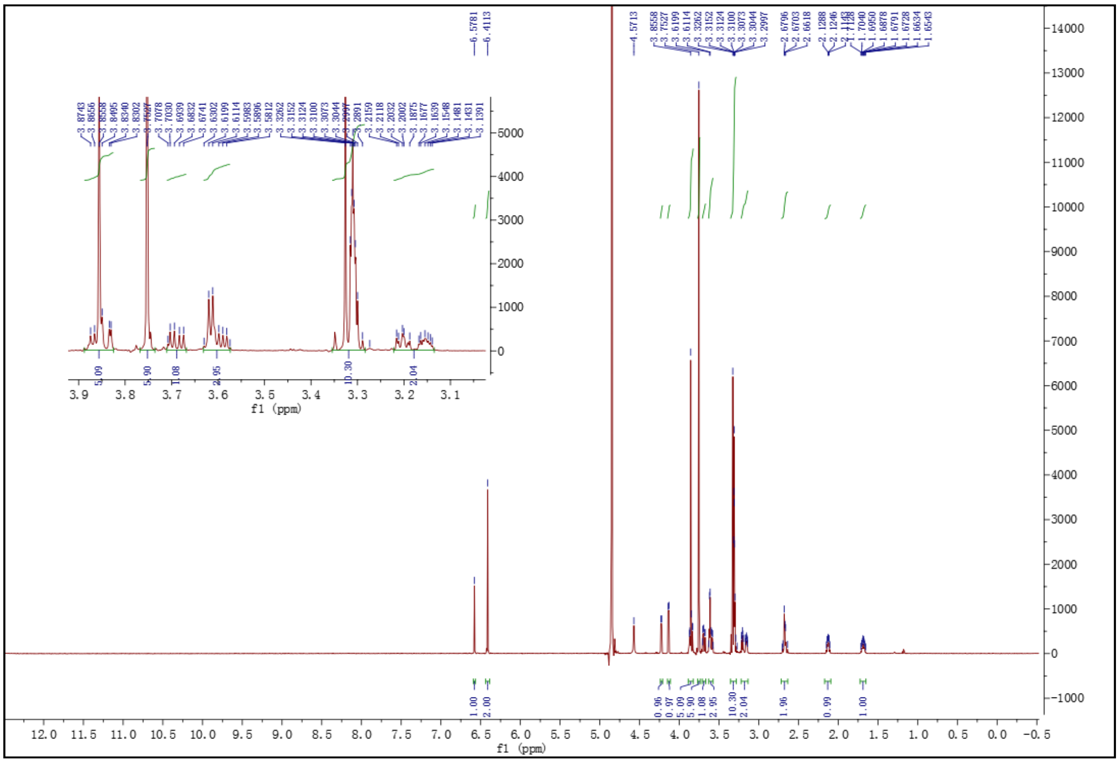

Figure 7b. ^{13}C-NMR spectrum (MeOD, 150 MHz) of compound **7**.

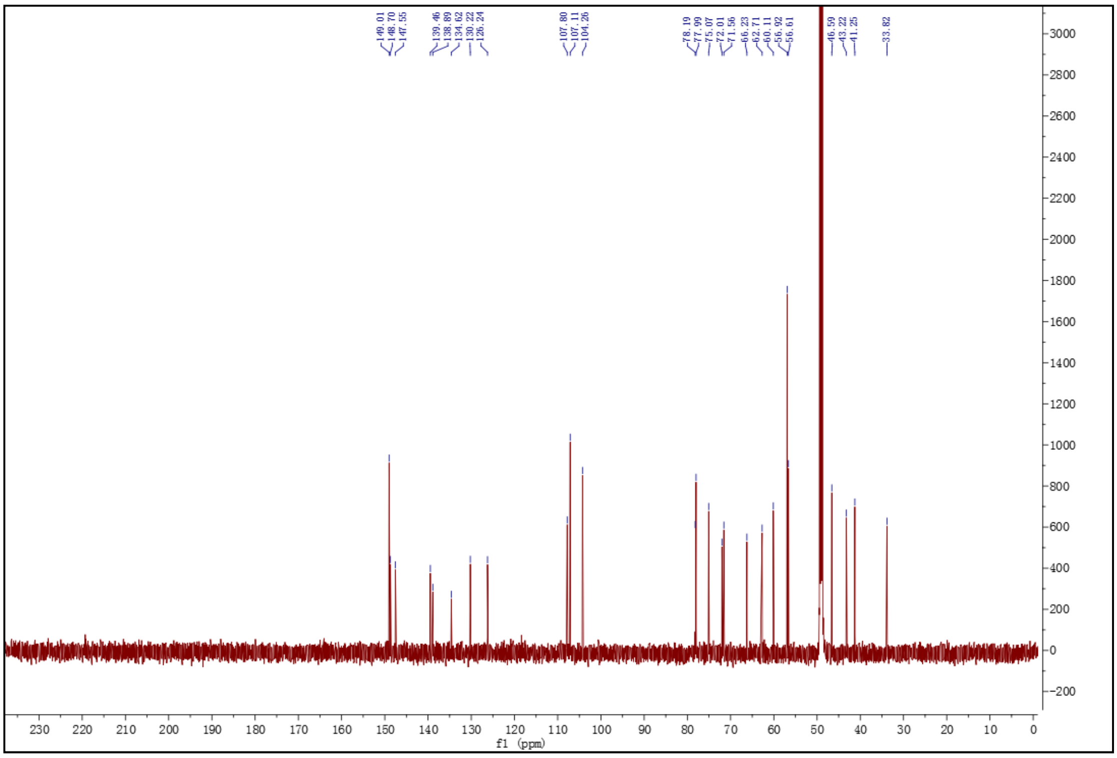

Figure 8a. ^{1}H-NMR spectrum (MeOD, 600 MHz) of compound **8**.

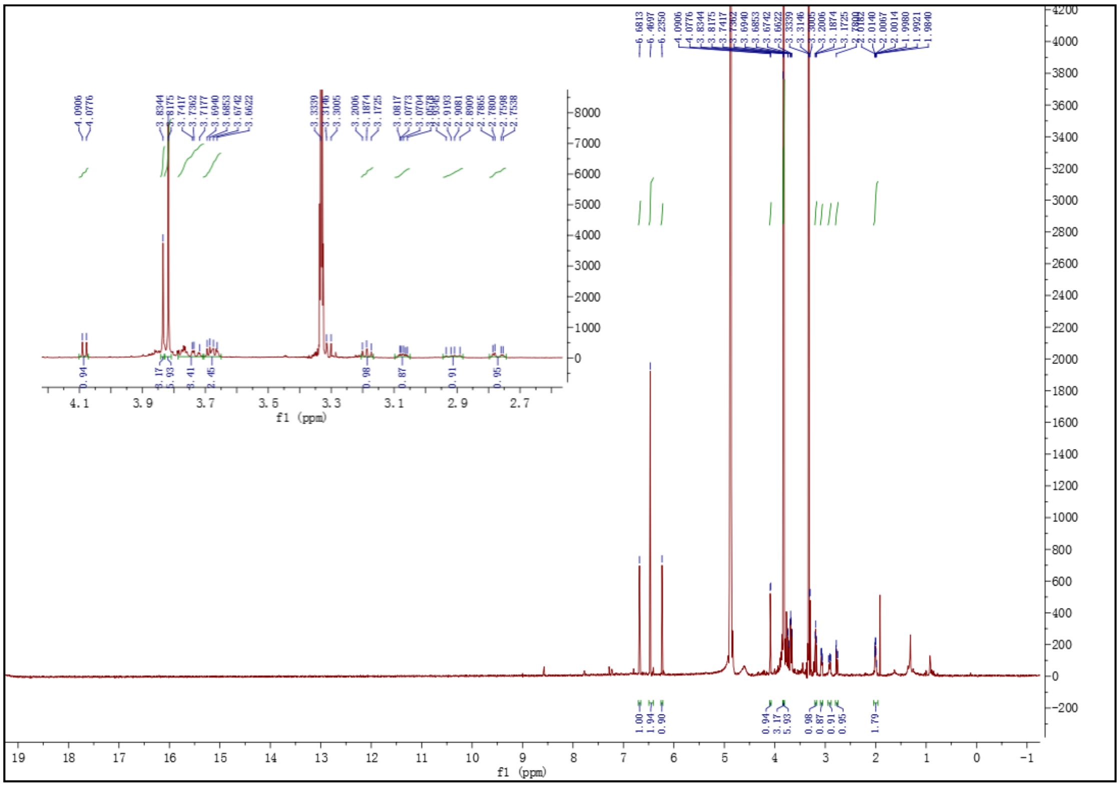

Figure 8b. ^{13}C-NMR spectrum (MeOD, 150 MHz) of compound **8**.

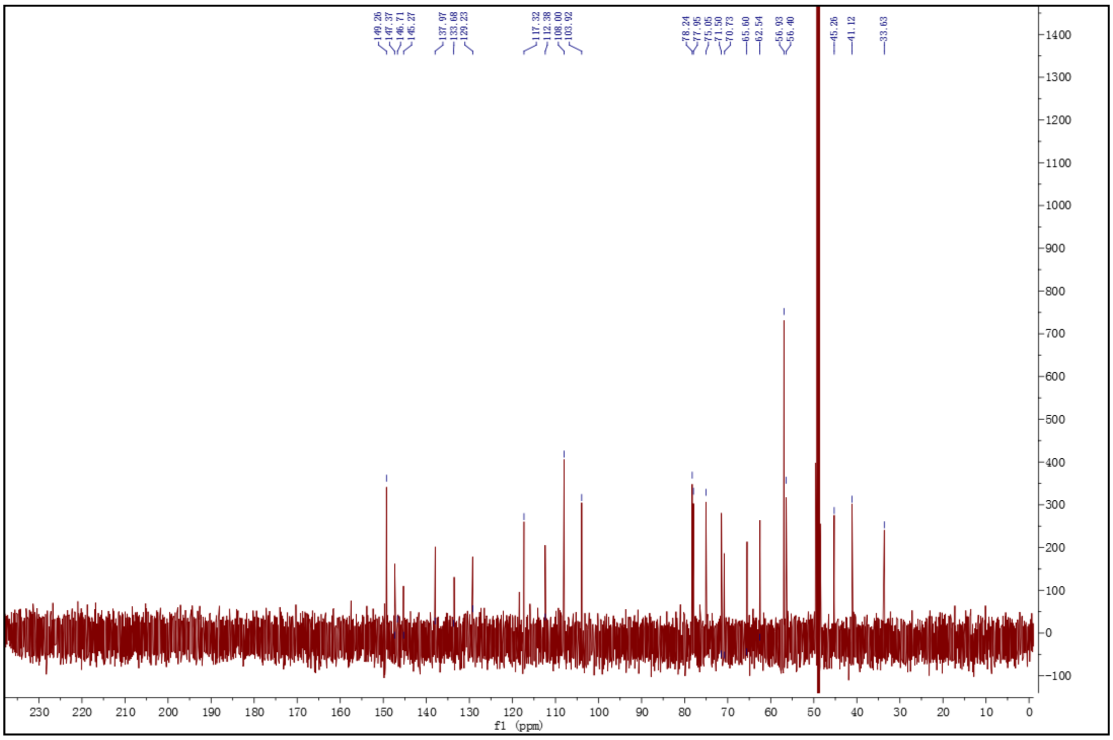

Figure 9a. ^{1}H-NMR spectrum (MeOD, 600 MHz) of compound **9**.

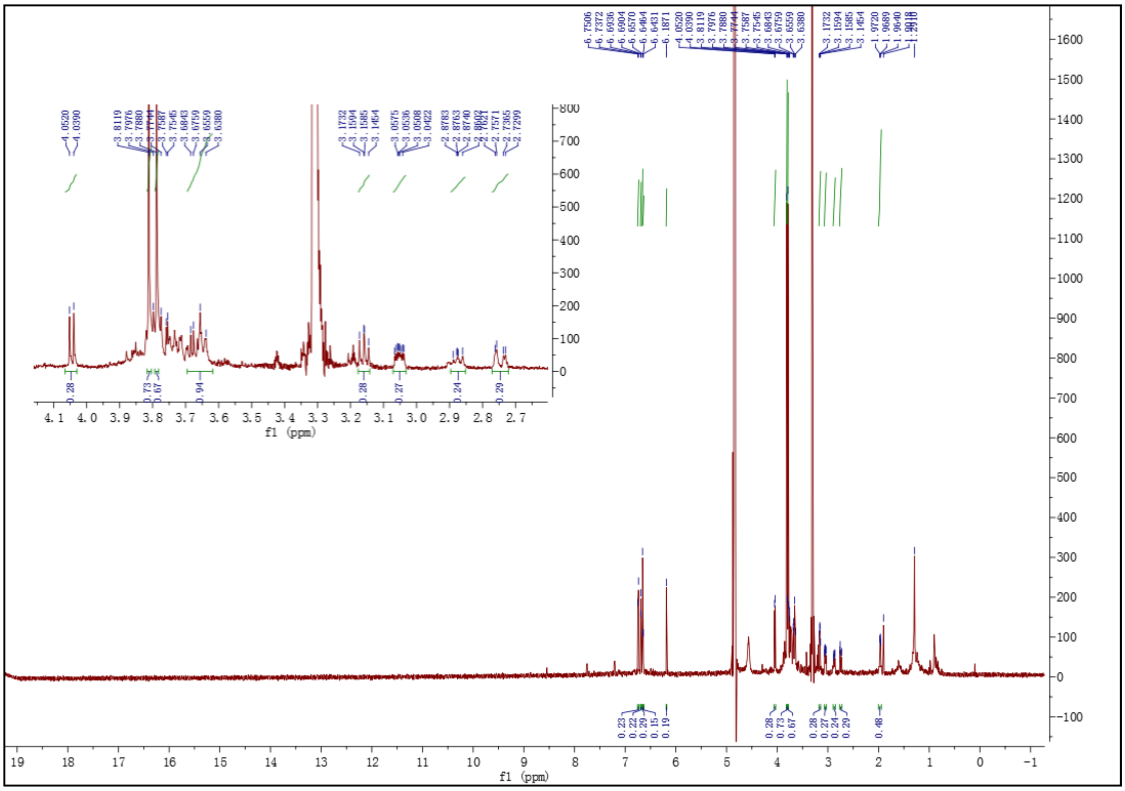

Figure 9b. ^{13}C-NMR spectrum (MeOD, 150 MHz) of compound **9**.

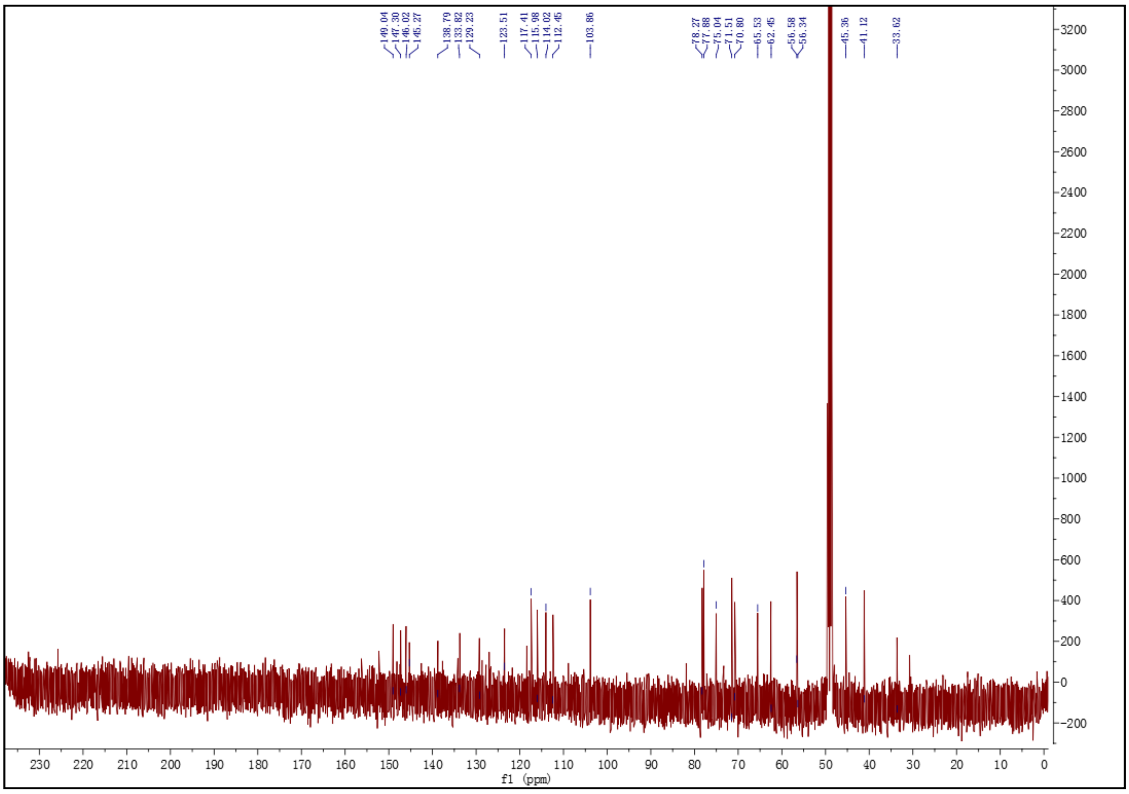

Figure 10a. ^{1}H-NMR spectrum (MeOD, 600 MHz) of compound **10**.

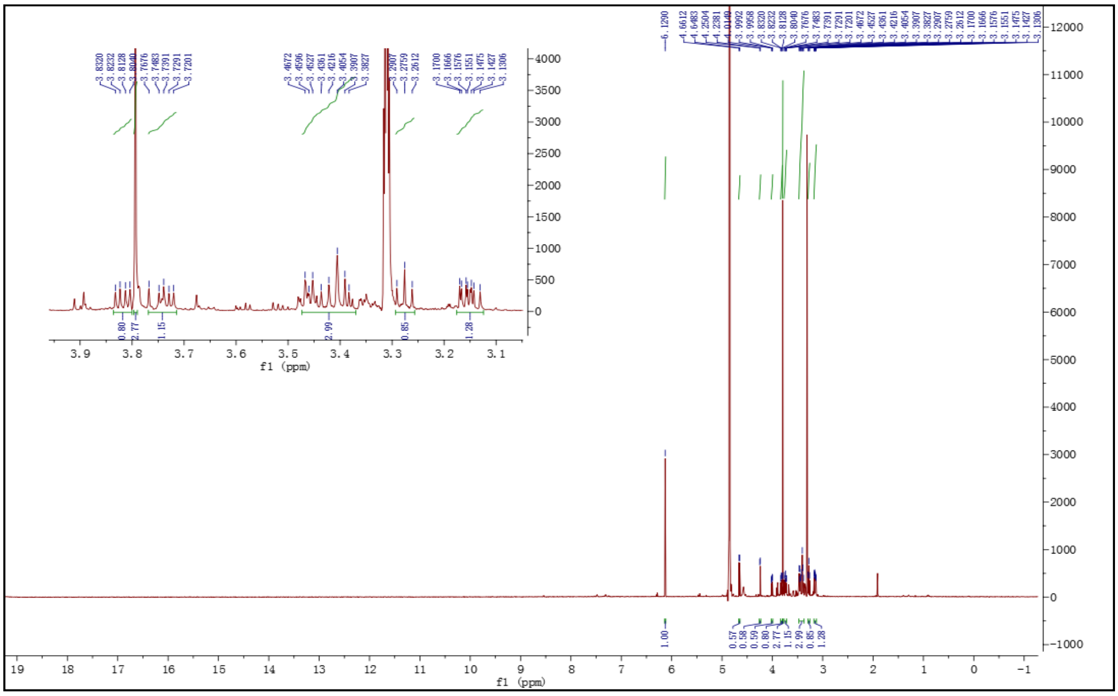

Figure 10b. ^{13}C-NMR spectrum (MeOD, 150 MHz) of compound **10**.

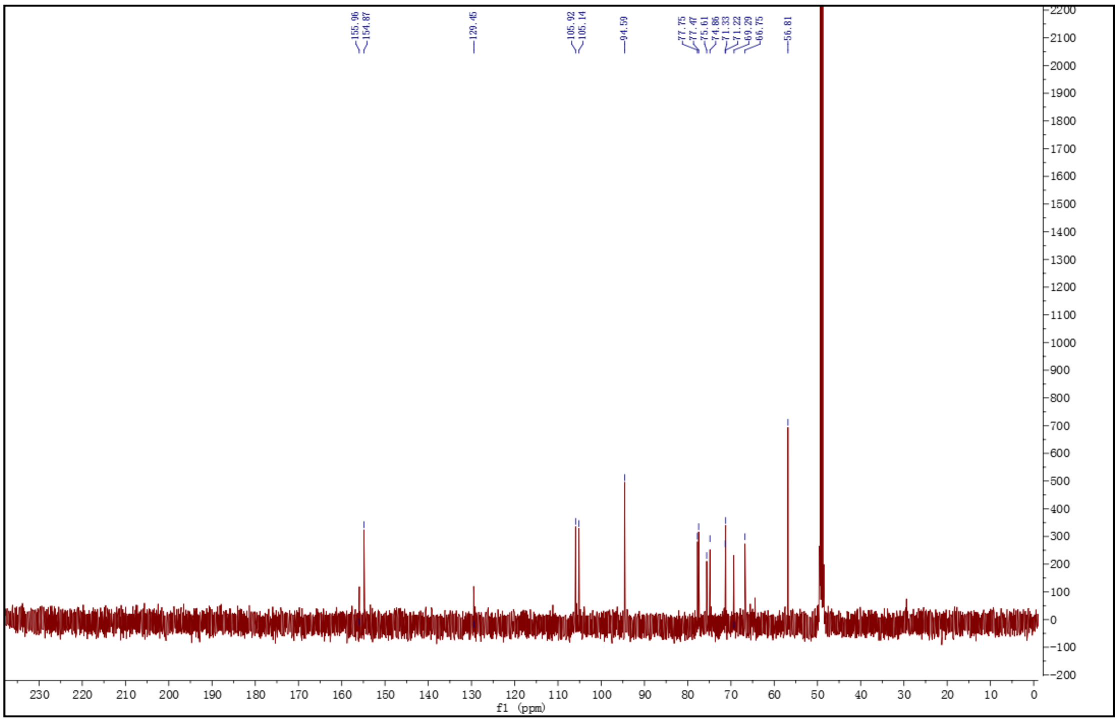

Figure 11a. ^{1}H-NMR spectrum (MeOD, 600 MHz) of compound **11**.

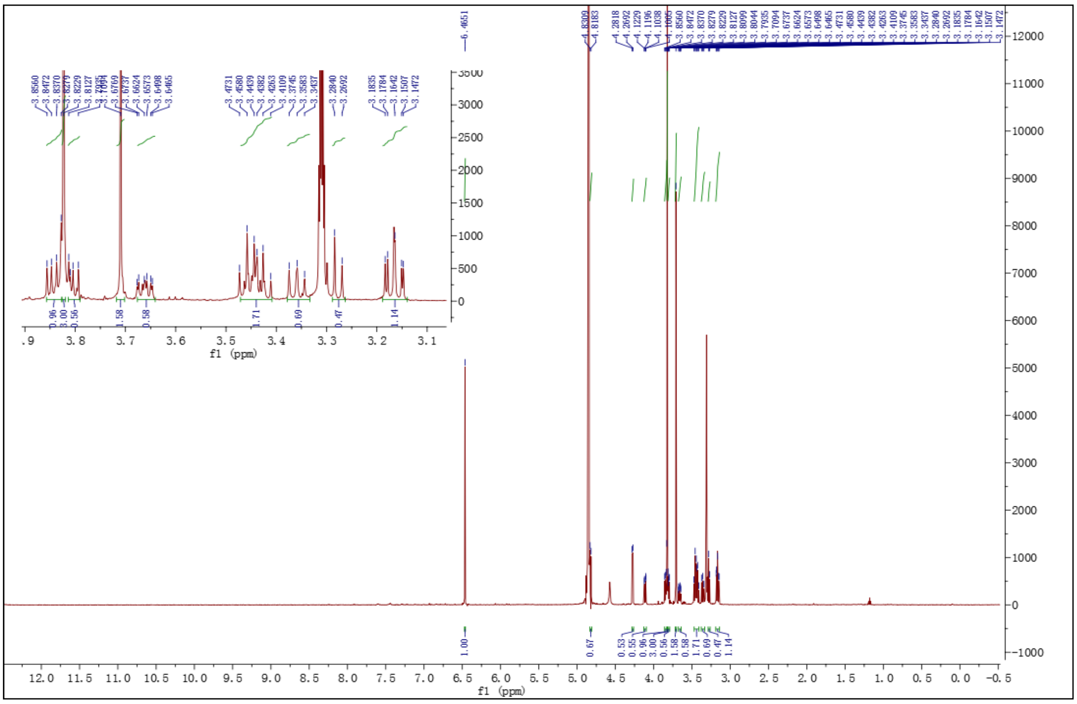

Figure 11b. ^{13}C-NMR spectrum (MeOD, 150 MHz) of compound **11**.

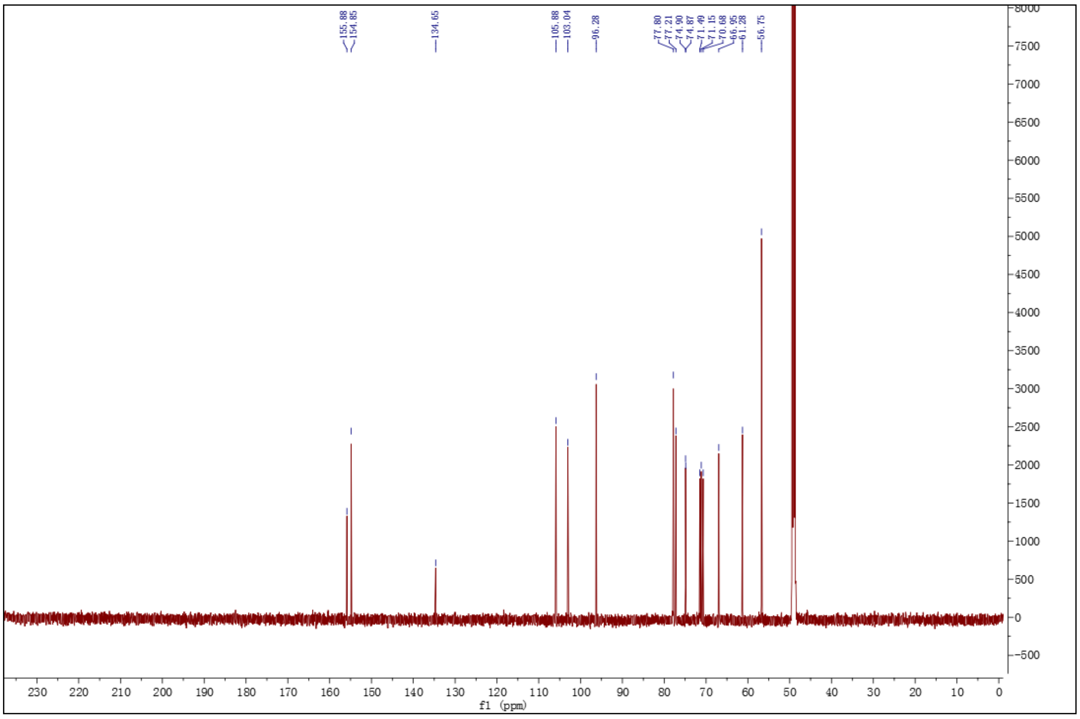

Figure 12a. ^{1}H-NMR spectrum (MeOD, 600 MHz) of compound **12**.

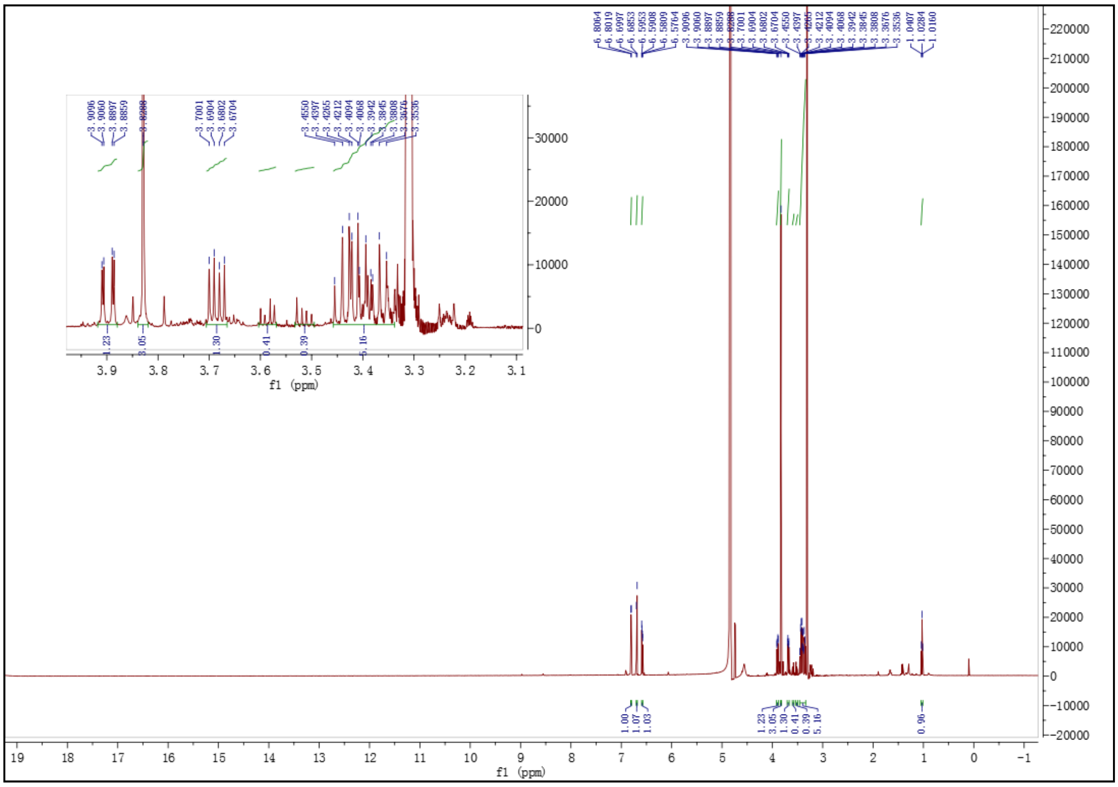

Figure 12b. ^{13}C-NMR spectrum (MeOD, 150 MHz) of compound **12**.

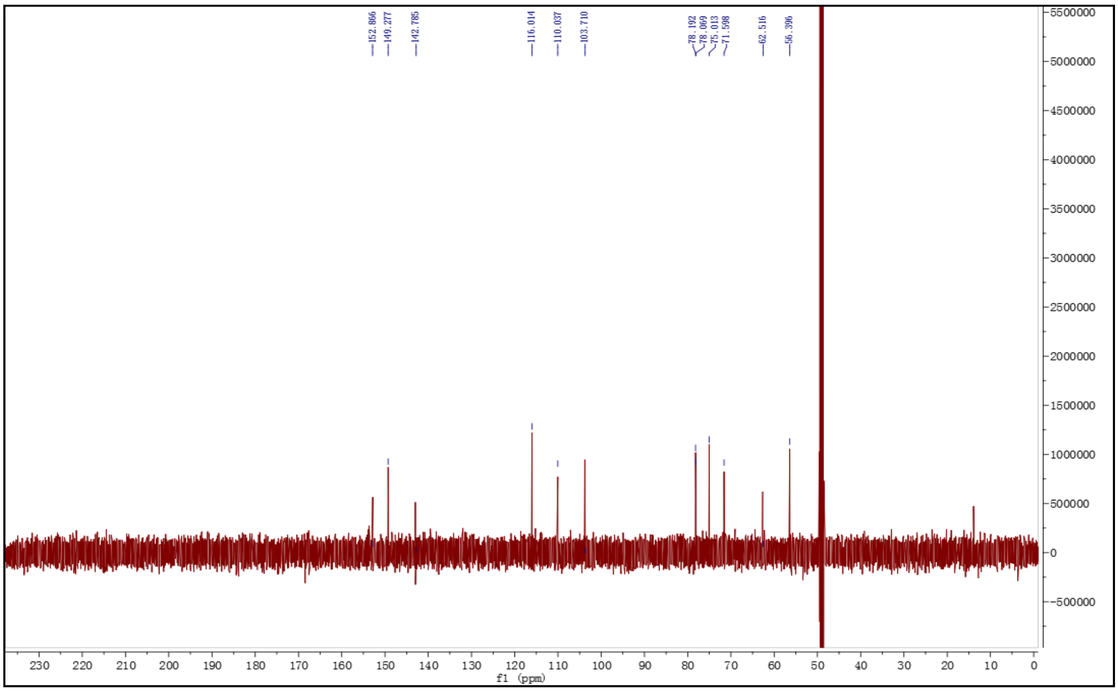

Figure 13a. ^{1}H-NMR spectrum (MeOD, 600 MHz) of compound **13**.

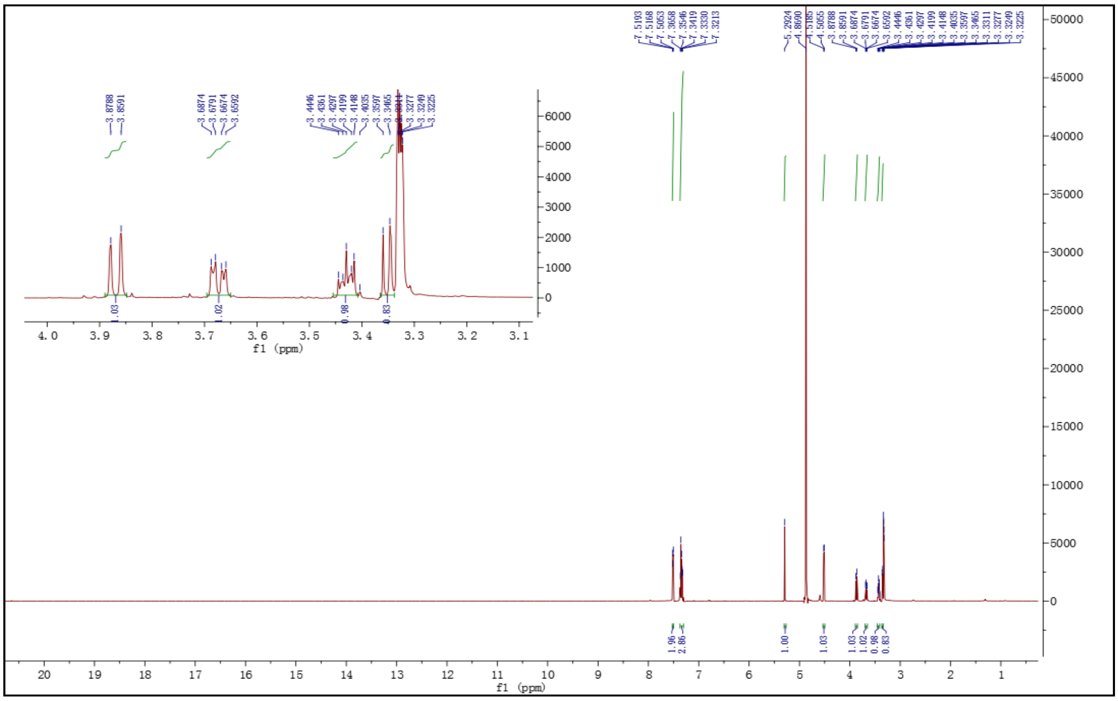

Figure 13b. ^{13}C-NMR spectrum (MeOD, 150 MHz) of compound **13**.

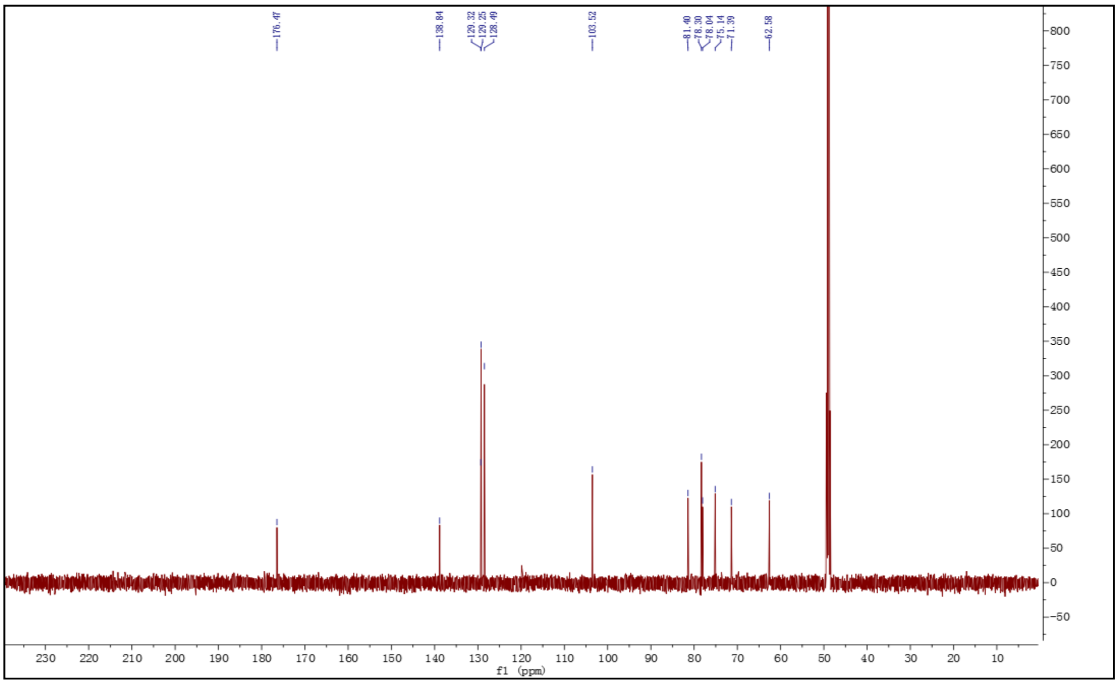

Figure 14a. ^{1}H-NMR spectrum (MeOD, 600 MHz) of compound **14**.

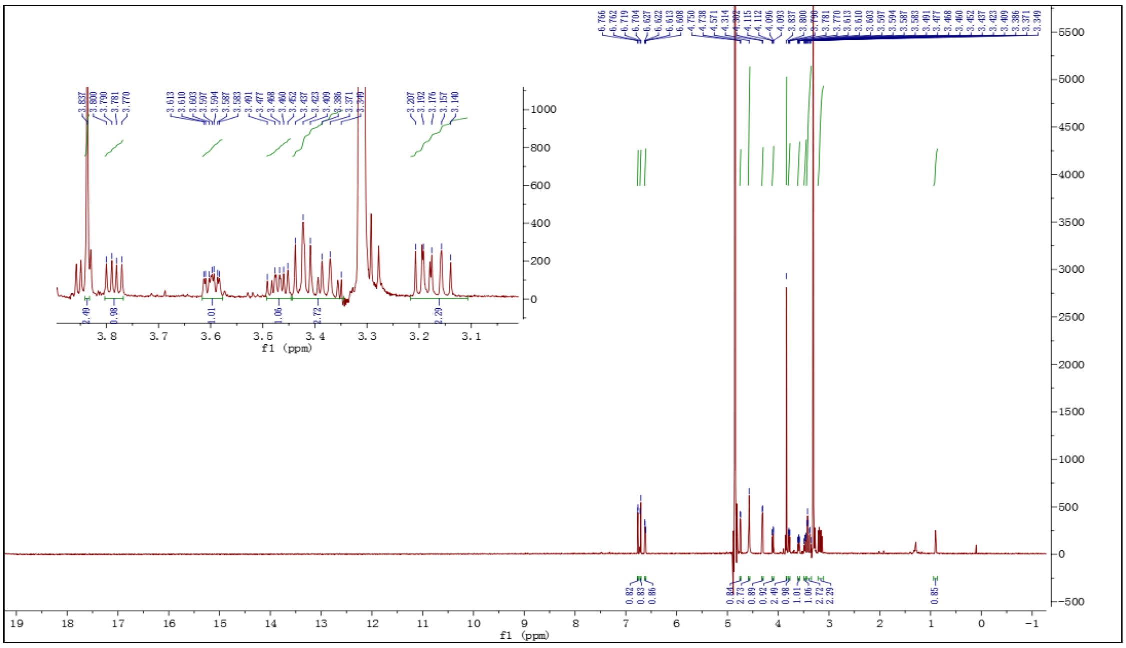

Figure 14b. ^{13}C-NMR spectrum (MeOD, 150 MHz) of compound **14**.

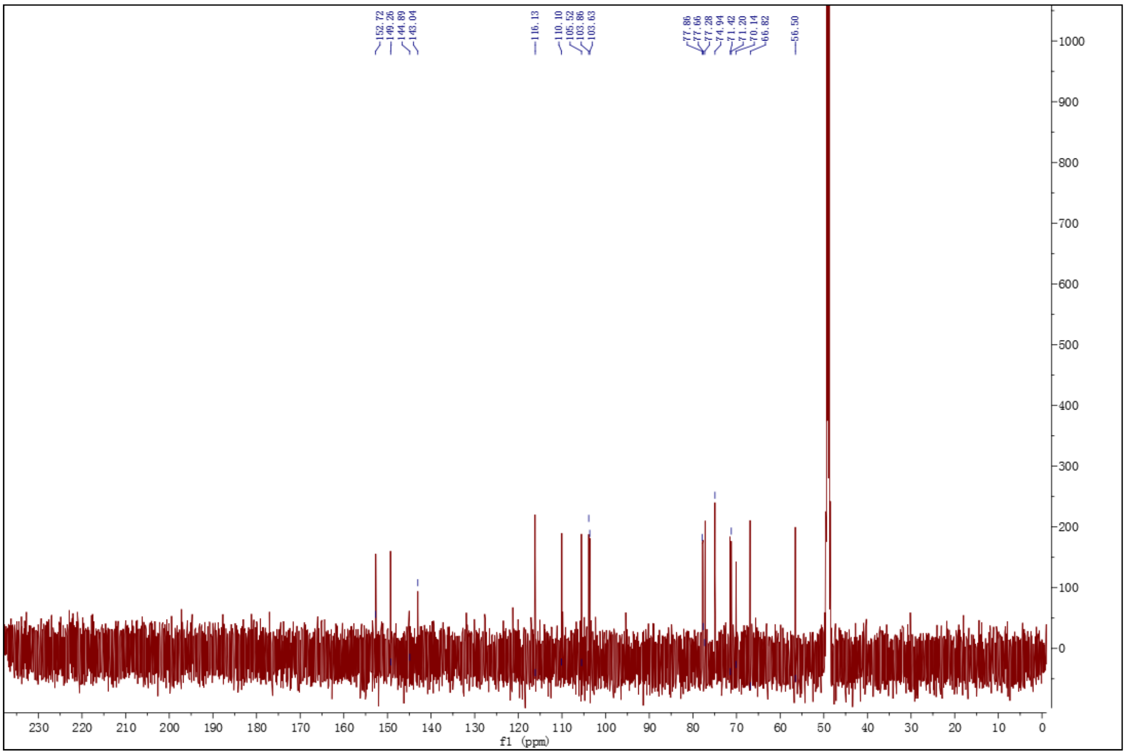

Figure 15a. ^{1}H-NMR spectrum (MeOD, 600 MHz) of compound **15**.

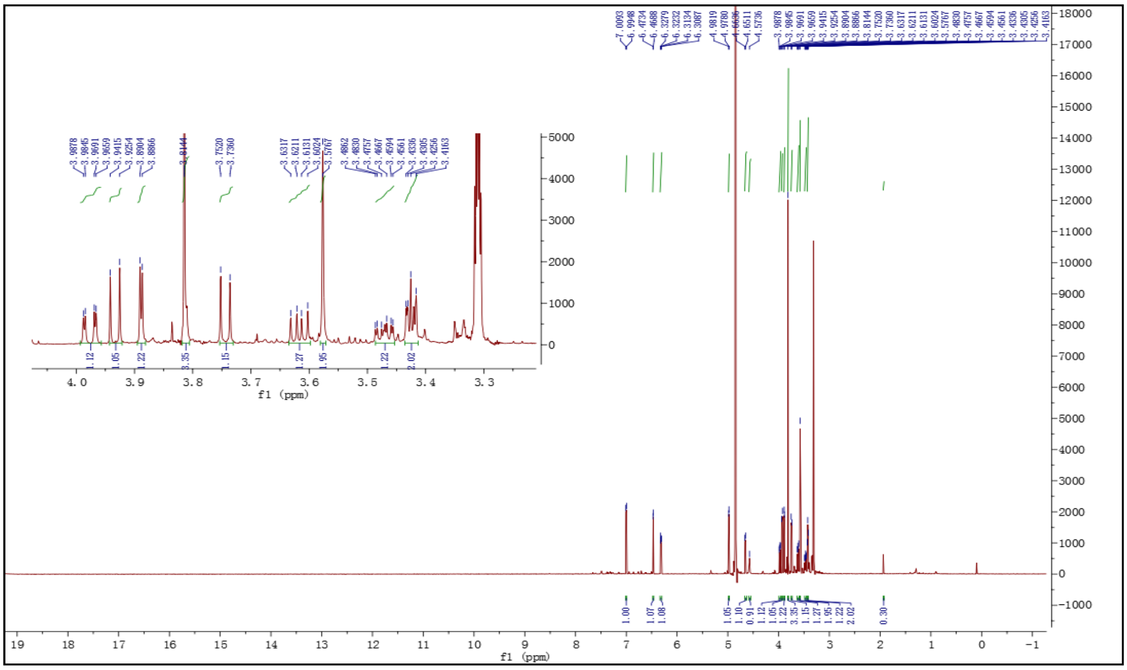

Figure 15b. ^{13}C-NMR spectrum (MeOD, 150 MHz) of compound **15**.

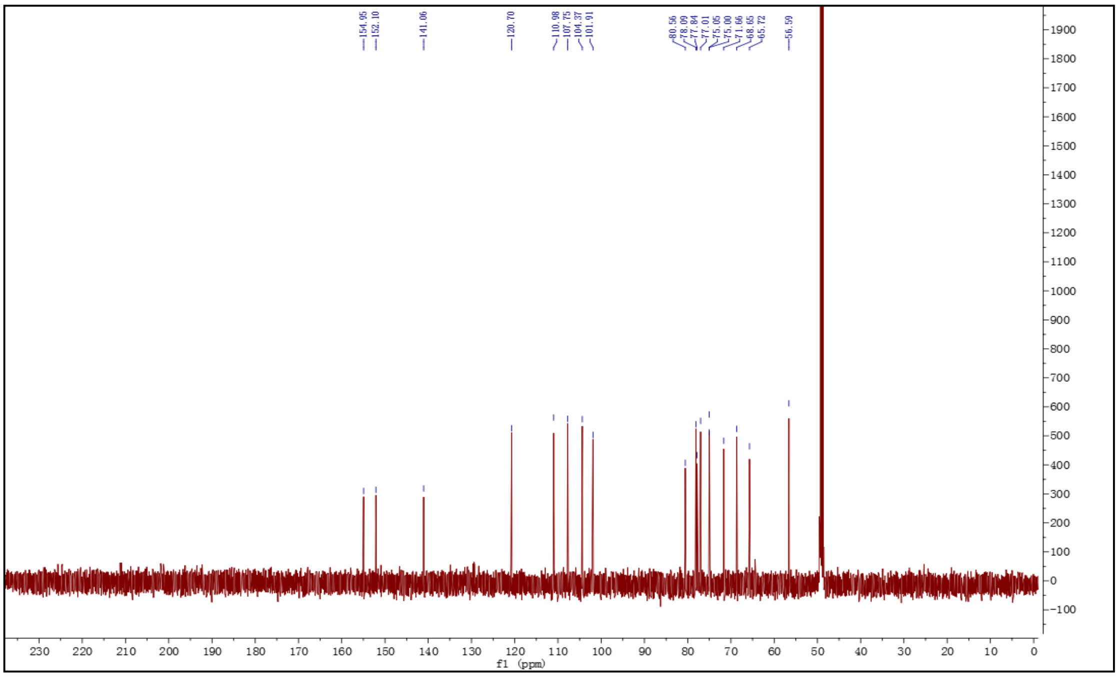

Figure 16. FTIR spectrum (KBr) of compound **1**.

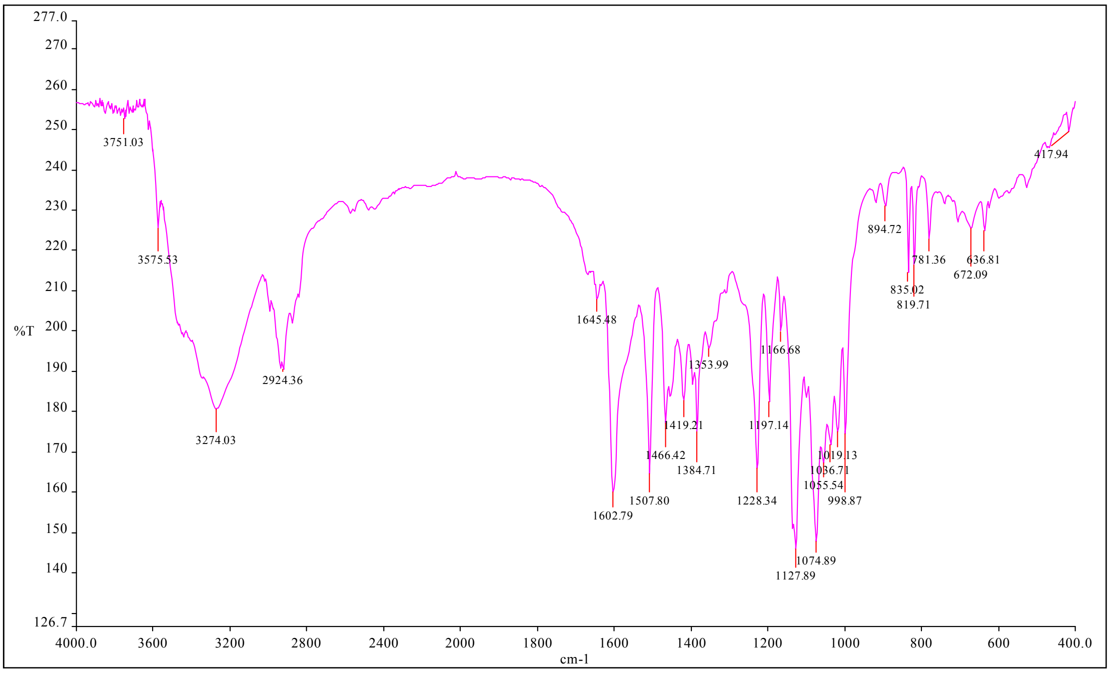

Figure 17. FTIR spectrum (KBr) of compound **2**.

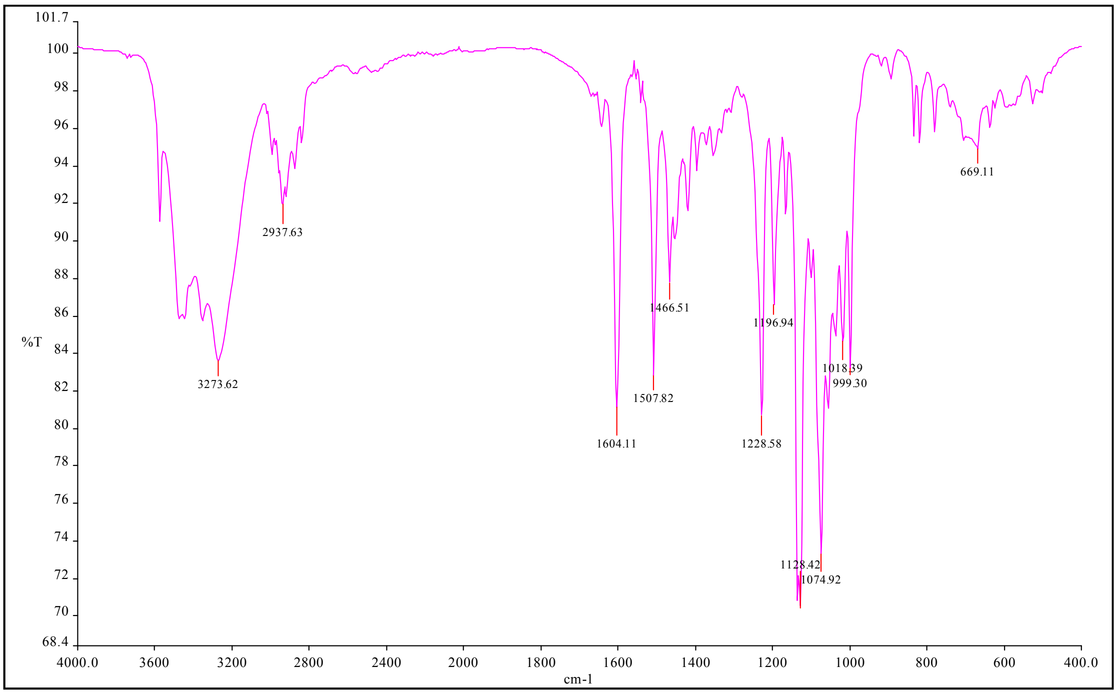

Figure 18. FTIR spectrum (KBr) of compound **3**.

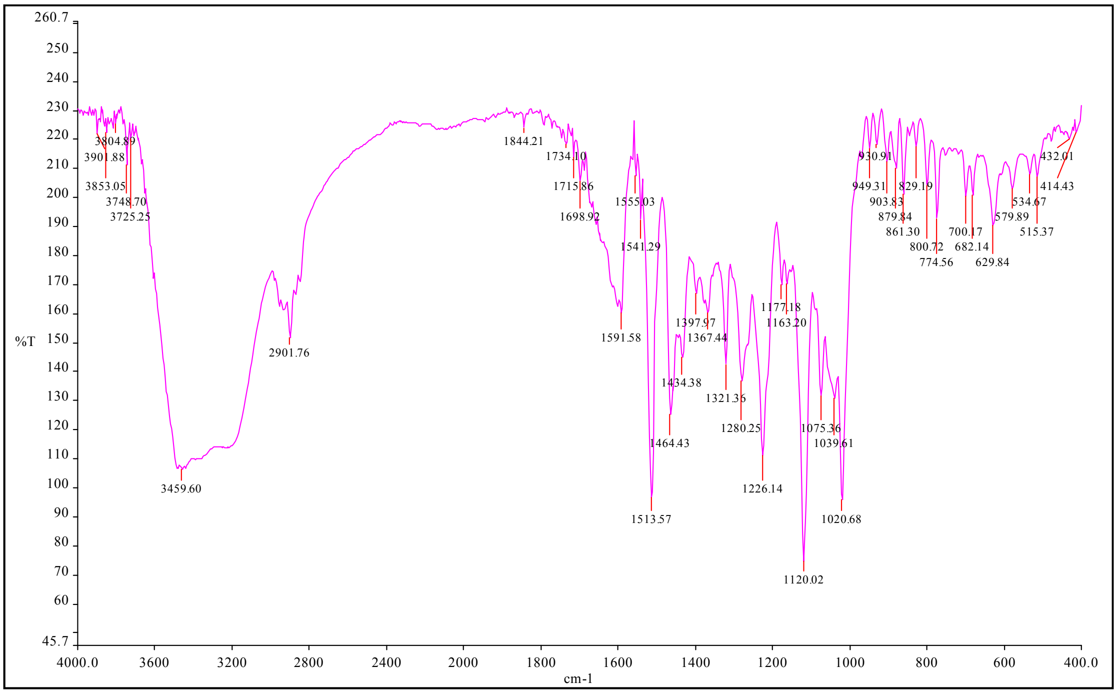

Figure 19. FTIR spectrum (KBr) of compound **4**.

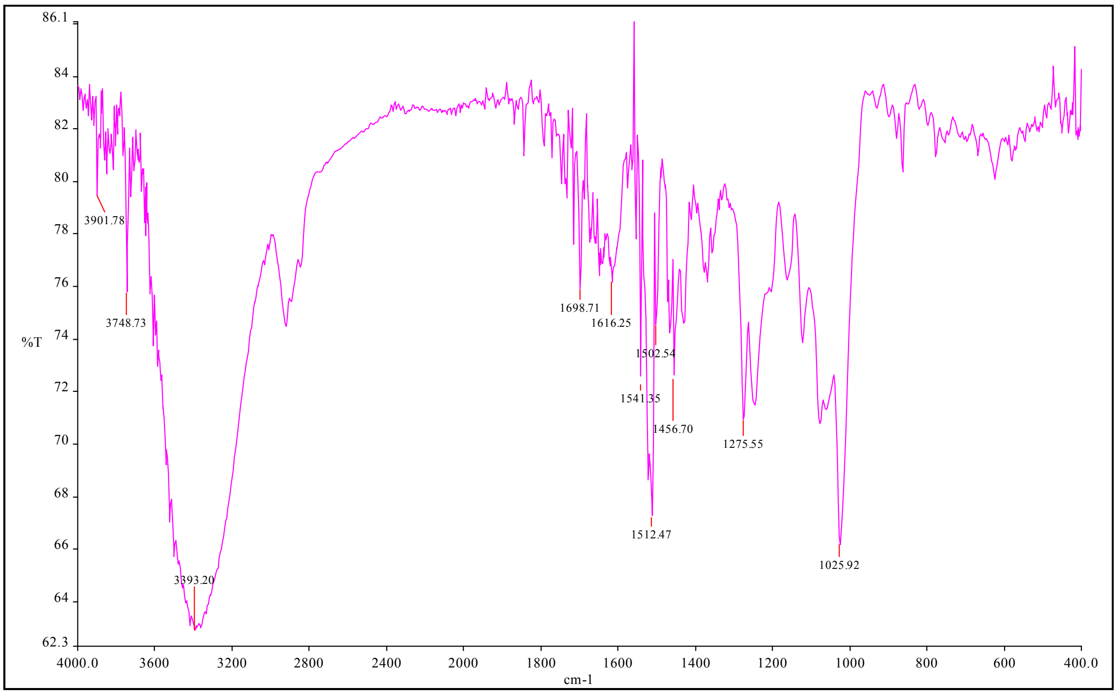

Figure 20. FTIR spectrum (KBr) of compound **5**.

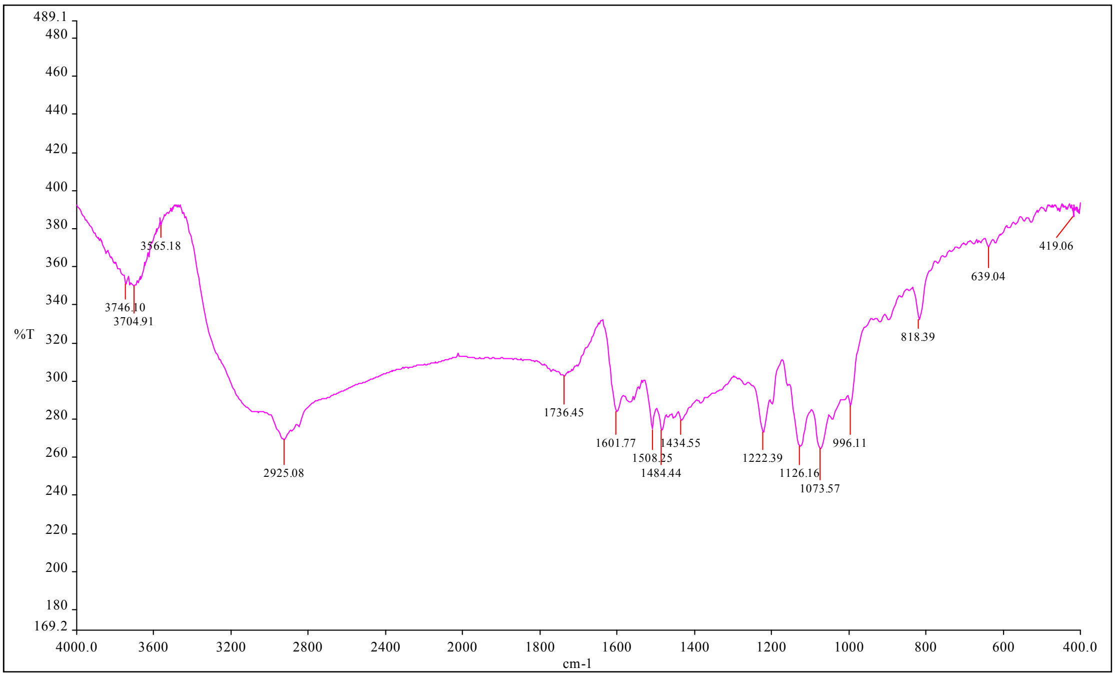

Figure 21. FTIR spectrum (KBr) of compound **6**.

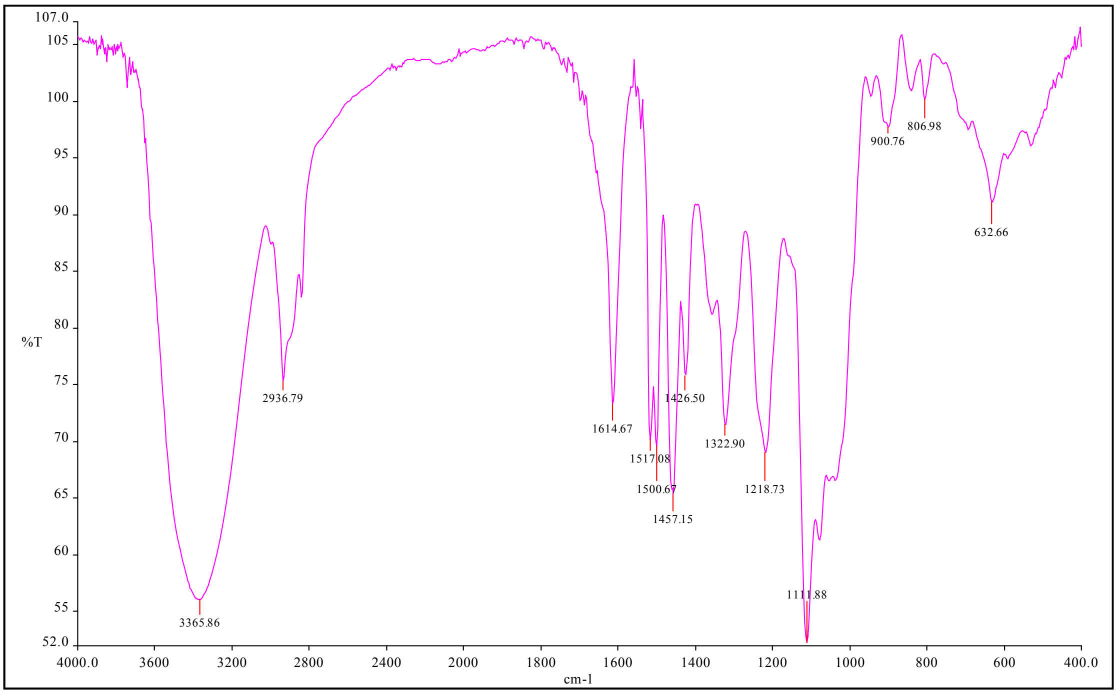

Figure 22. FTIR spectrum (KBr) of compound **7**.

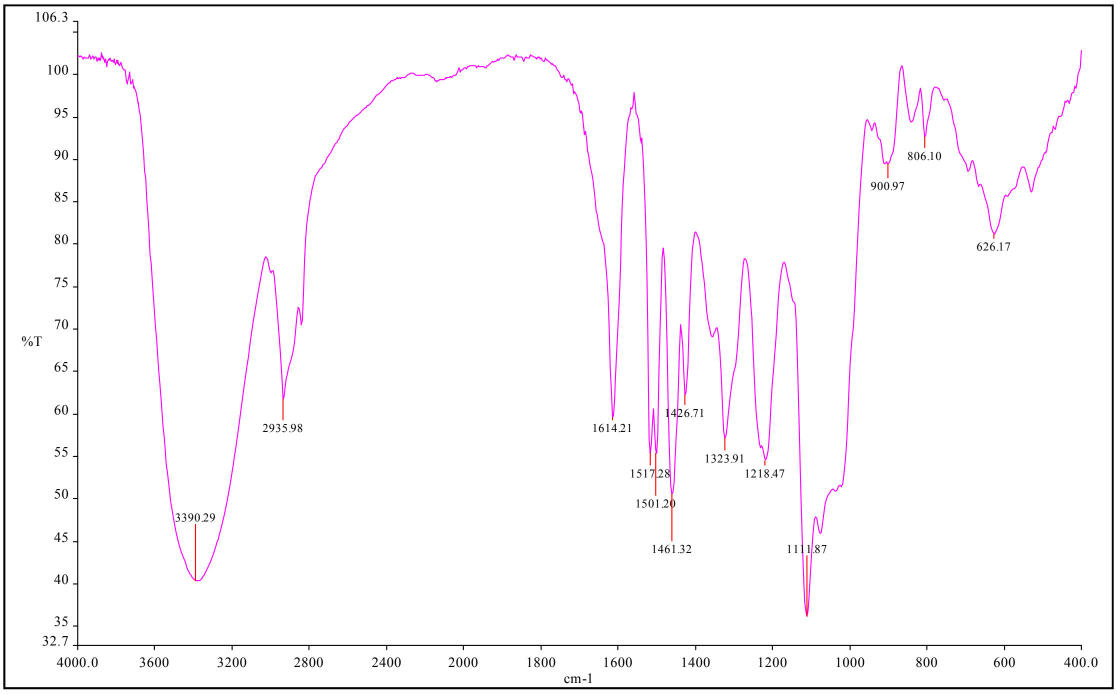

Figure 23. FTIR spectrum (KBr) of compound **8**.

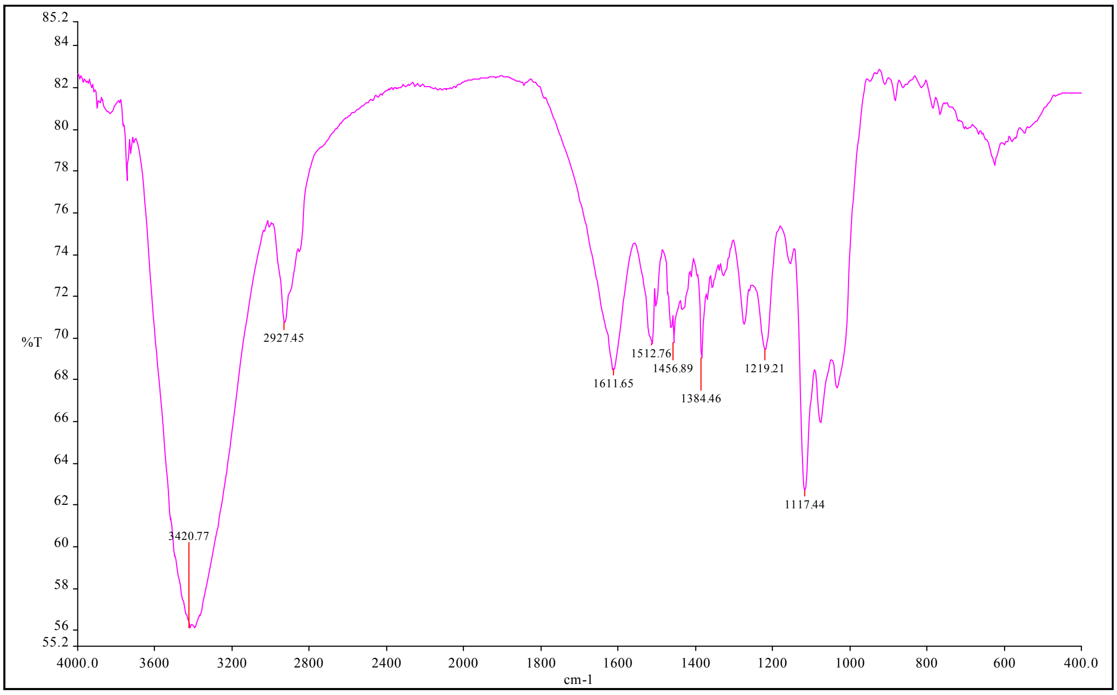

Figure 24. FTIR spectrum (KBr) of compound **9**.

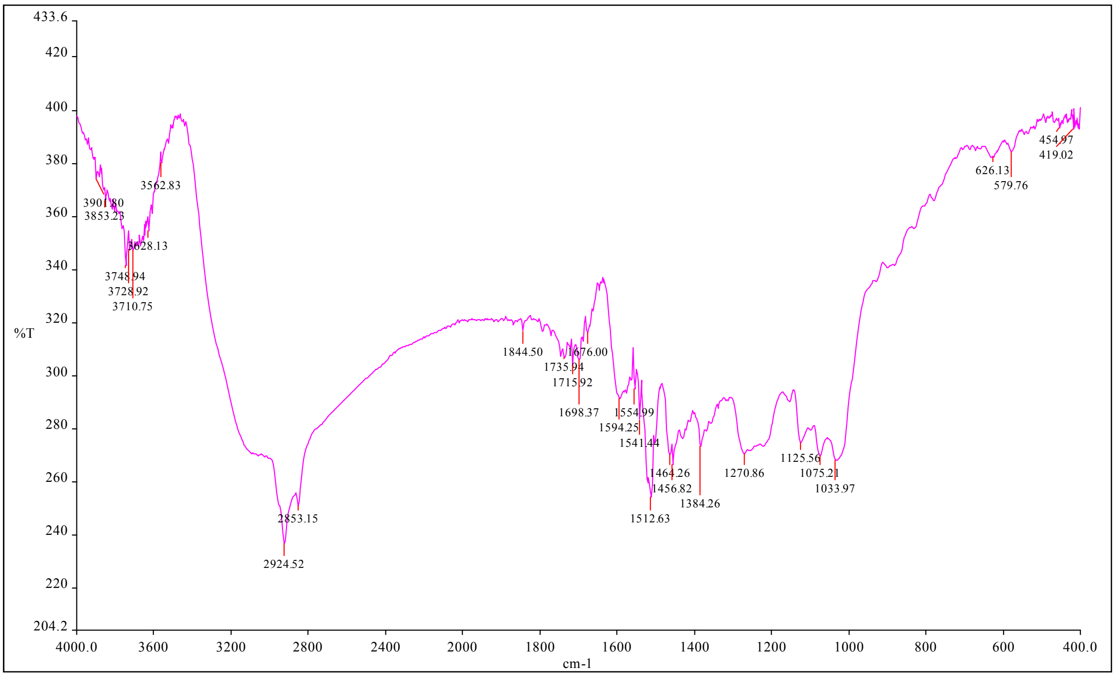

Figure 25. FTIR spectrum (KBr) of compound **10**.

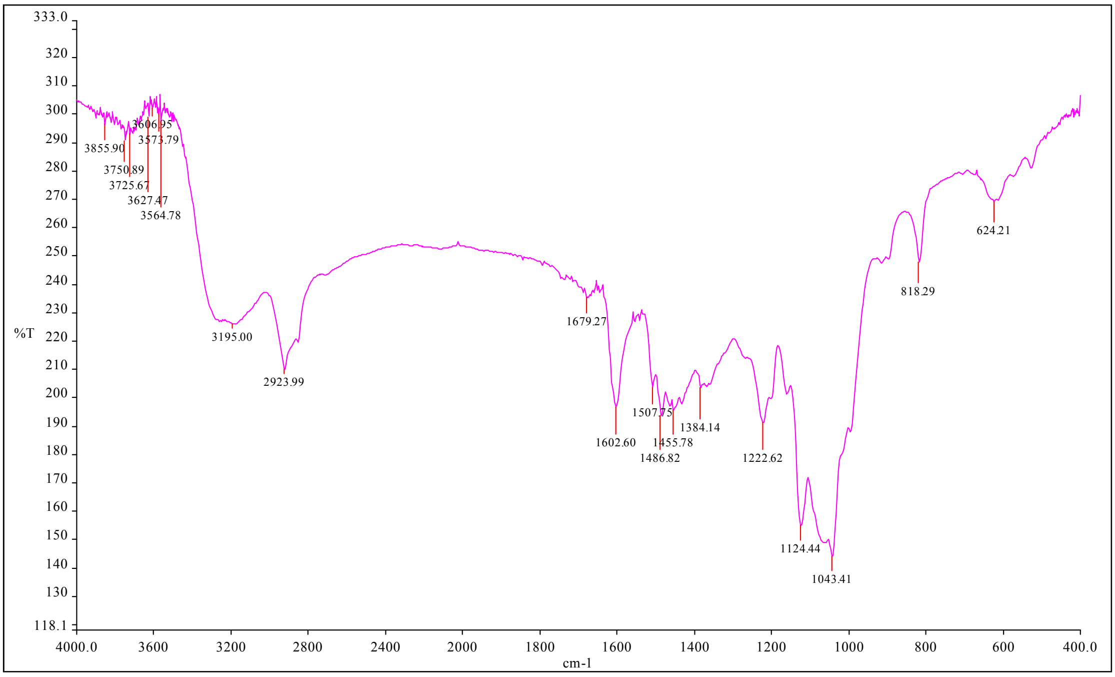

Figure 26. FTIR spectrum (KBr) of compound **11**.

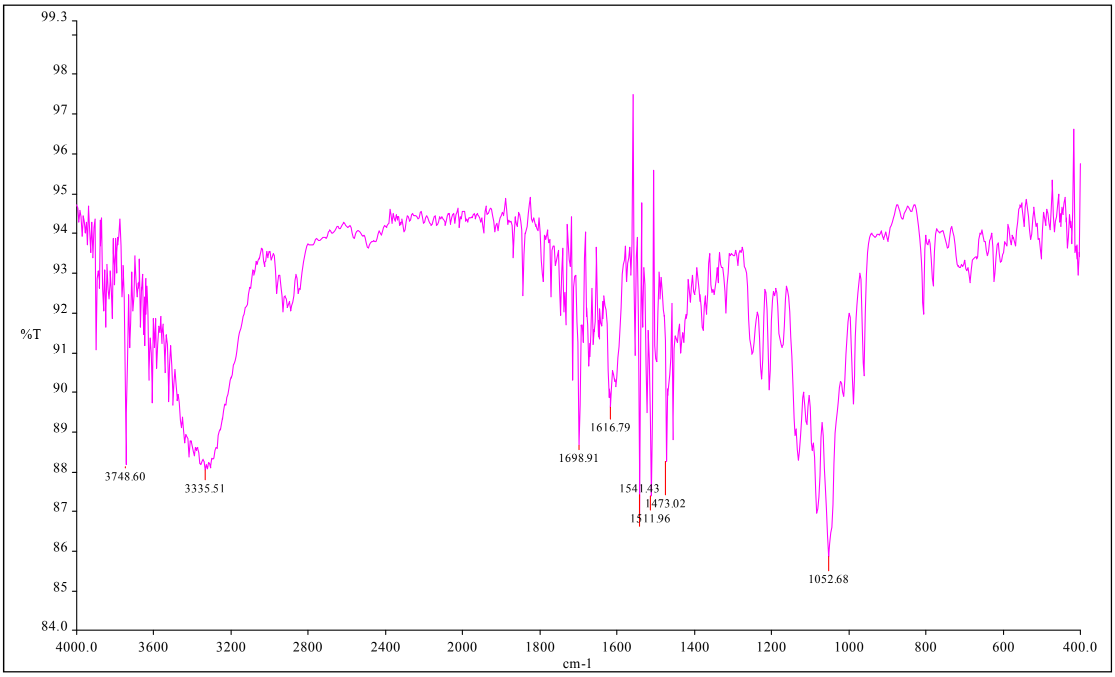

Figure 27. FTIR spectrum (KBr) of compound **12**.

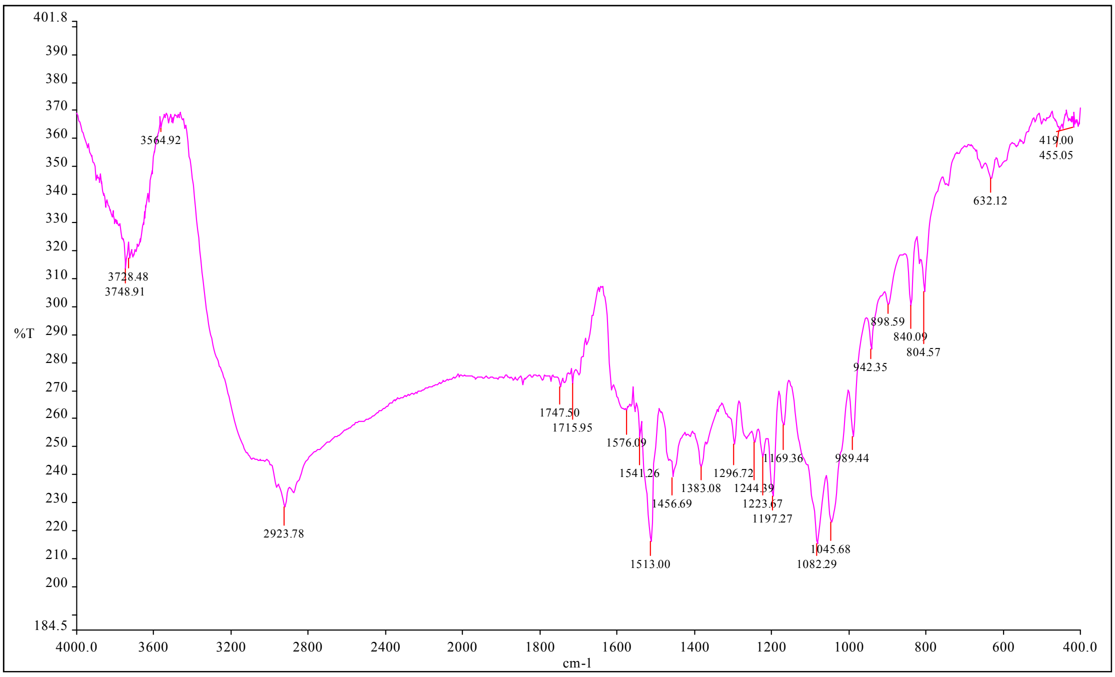

Figure 28. FTIR spectrum (KBr) of compound **13**.

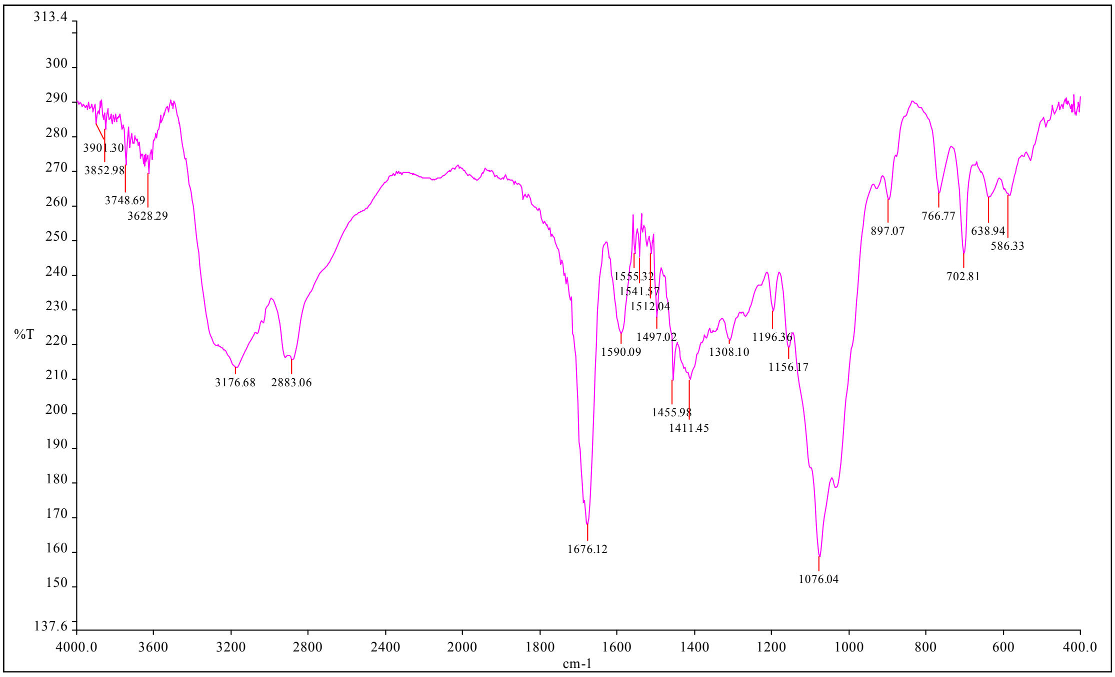

Figure 29. FTIR spectrum (KBr) of compound **14**.

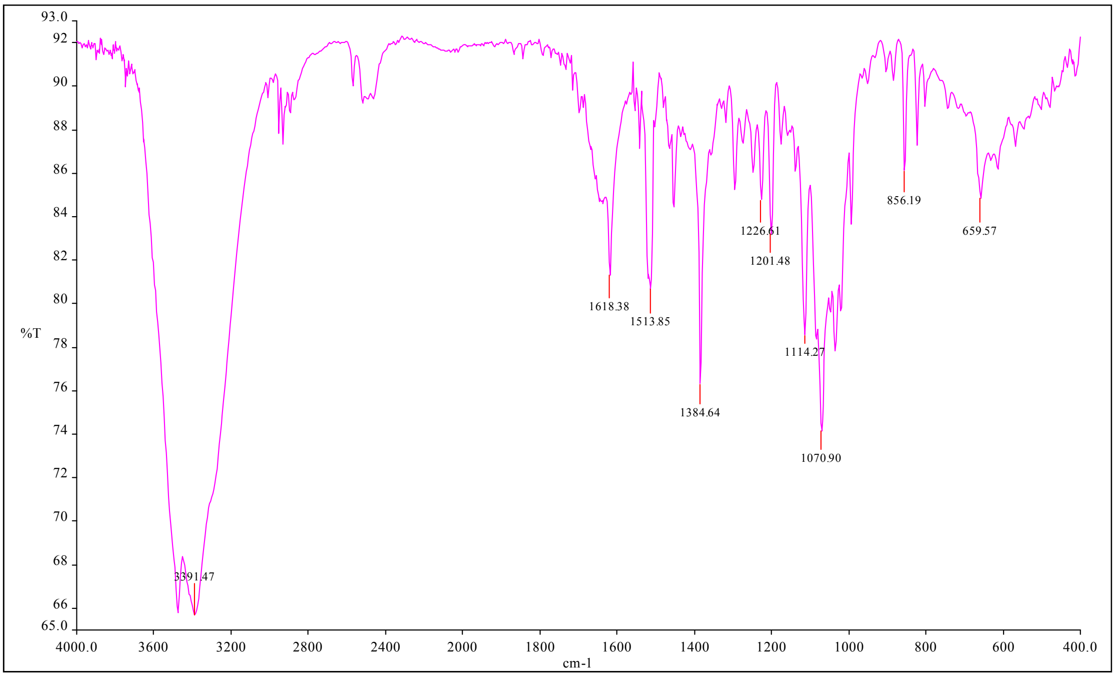

Figure 30. FTIR spectrum (KBr) of compound **15**.

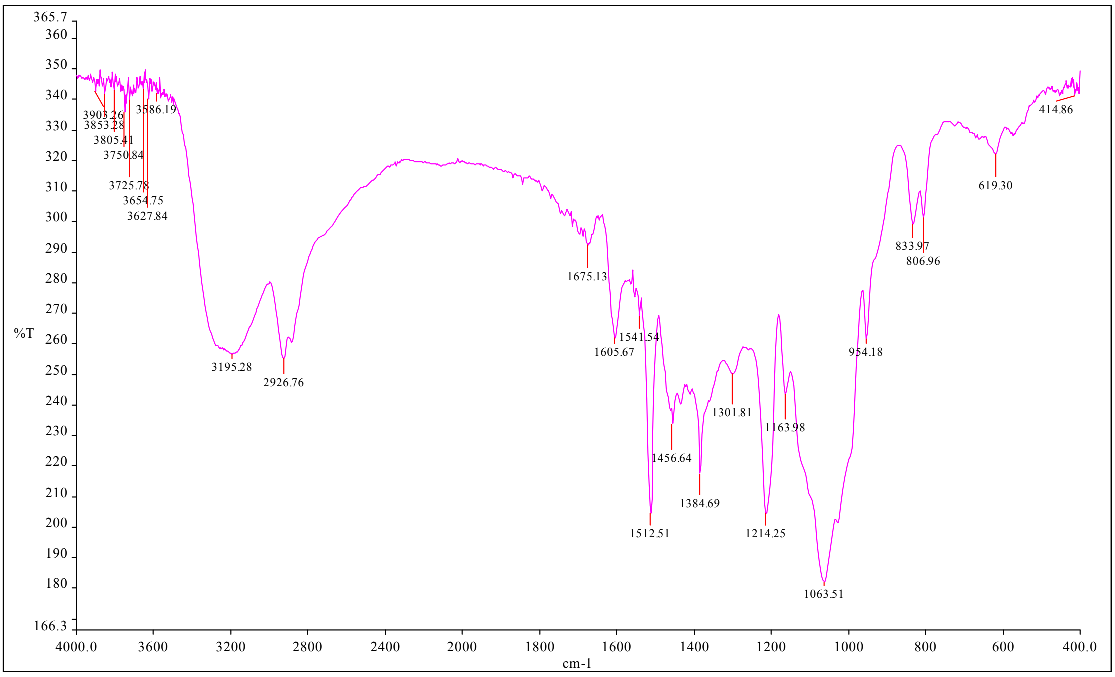

Supplement: Supplementary file 1 [file molecules-17-12330-s001.pdf]
